# NANOG reprograms prostate cancer cells to castration resistance via dynamically repressing and engaging the AR/FOXA1 signaling axis

**DOI:** 10.1038/celldisc.2016.41

**Published:** 2016-11-15

**Authors:** Collene R Jeter, Bigang Liu, Yue Lu, Hsueh-Ping Chao, Dingxiao Zhang, Xin Liu, Xin Chen, Qiuhui Li, Kiera Rycaj, Tammy Calhoun-Davis, Li Yan, Qiang Hu, Jianmin Wang, Jianjun Shen, Song Liu, Dean G Tang

**Affiliations:** 1 Department of Epigenetics and Molecular Carcinogenesis, The University of Texas MD Anderson Cancer Center, Smithville, TX, USA; 2 Department of Pharmacology and Therapeutics, Roswell Park Cancer Institute, Buffalo, NY, USA; 3 Department of Biostatistics and Bioinformatics, Roswell Park Cancer Institute, Buffalo, NY, USA; 4 Cancer Stem Cell Institute, Research Center for Translational Medicine, East Hospital, Tongji University School of Medicine, Shanghai, China; 5 Centers for Cancer Epigenetics, Stem Cell and Developmental Biology, RNA Interference and Non-coding RNAs and Molecular Carcinogenesis, University of Texas MD Anderson Cancer Center, Houston, TX, USA

**Keywords:** AR, cancer stem cells, castration resistance, FOXA1, NANOG, prostate cancer

## Abstract

The pluripotency transcription factor NANOG has been implicated in tumor development, and NANOG-expressing cancer cells manifest stem cell properties that sustain tumor homeostasis, mediate therapy resistance and fuel tumor progression. However, how NANOG converges on somatic circuitry to trigger oncogenic reprogramming remains obscure. We previously reported that inducible NANOG expression propels the emergence of aggressive castration-resistant prostate cancer phenotypes. Here we first show that endogenous NANOG is required for the growth of castration-resistant prostate cancer xenografts. Genome-wide chromatin immunoprecipitation sequencing coupled with biochemical assays unexpectedly reveals that NANOG co-occupies a distinctive proportion of androgen receptor/Forkhead box A1 genomic loci and physically interacts with androgen receptor and Forkhead box A1. Integrative analysis of chromatin immunoprecipitation sequencing and time-resolved RNA sequencing demonstrates that NANOG dynamically alters androgen receptor/Forkhead box A1 signaling leading to both repression of androgen receptor-regulated pro-differentiation genes and induction of genes associated with cell cycle, stem cells, cell motility and castration resistance. Our studies reveal global molecular mechanisms whereby NANOG reprograms prostate cancer cells to a clinically relevant castration-resistant stem cell-like state driven by distinct NANOG-regulated gene clusters that correlate with patient survival. Thus, reprogramming factors such as NANOG may converge on and alter lineage-specific master transcription factors broadly in somatic cancers, thereby facilitating malignant disease progression and providing a novel route for therapeutic resistance.

## Introduction

Cancers are immortal at the population level, sustained and driven by stem-like cancer cells expressing immortality or self-renewal molecules. The pluripotency transcription factor (TF) NANOG is aberrantly expressed in a spectrum of cancers, including germ cell tumors and cancers of the brain, head and neck, colon, breast, ovary, liver and prostate, among others [1, 2]. Somatic cancer-specific NANOG has been convincingly demonstrated to arise predominantly from a highly similar (harboring a single conserved amino-acid change) retrogene variant located on chromosome 15 and termed *NANOGP8* [3–6], although expression from the parental *NANOG1* locus has also been reported [6–8]. We have shown that prostate cancer (PCa)-associated NANOG is derived primarily from *NANOGP8* and is enriched in CD44^+^ PCa stem/progenitor cells, and inversely correlates with differentiation factors androgen receptor (AR) and prostate-specific antigen (PSA) [3, 9].

Mirroring NANOG’s role in the maintenance of renewing embryonic stem cells (ESCs), NANOG’s expression in cancers correlates with pathophysiological manifestations often attributed to the presence of tumor-initiating and tumor-propagating cancer cells phenotypically resembling stem cells, that is, cancer stem cells (CSCs) [10]. For example, functional assays have implicated NANOG as a key regulator of clonogenic growth, as well as tumorigenesis, therapy resistance and migration/metastasis in many cancers [1, 2]. Indeed, NANOG knockdown inhibits sphere formation, clonal growth, cell proliferation and tumor regeneration in breast, colon and prostate cancer cells [3] and NANOG knockdown in the undifferentiated, self-renewing and castration-resistant PSA^−/lo^ LAPC9 PCa cells inhibits tumor regeneration in androgen-deficient hosts [11]. Conversely, NANOG overexpression promotes CSC traits in many cancer cells and, importantly, castration-resistant tumor development in androgen-sensitive LNCaP PCa cells [9].

An important outstanding question is how tumor-specific retrogene NANOGP8, at the molecular level, promotes and maintains these tumorigenic and CSC traits in cancer cells. Here we address this critical question by performing genome-wide chromatin immunoprecipitation sequencing (ChIP-Seq) and transcriptome (that is, RNA sequencing (RNA-Seq)) analyses in LNCaP cells—a well-differentiated PCa cell line previously shown to harbor an androgen-independent (AI), self-renewing, stem-like cell subset [12]—modified to express a doxycycline (DOX)-inducible NANOGP8 transgene [9]. We show that NANOGP8 reprograms LNCaP cells to castration resistance by dynamically antagonizing and engaging AR/Forkhead box A1 (FOXA1) signaling as well as by engaging MYC signaling. Further substantiated by a spectrum of biological and biochemical assays, the broad applicability of these unexpected findings to human prostate carcinoma is demonstrated by a functional requirement for NANOG in xenograft models (LAPC4 and LAPC9) and by the observation that NANOG-regulated gene expression programs correlate with human patient transcriptomes and predict survival.

## Results

### Endogenous NANOG is required for castration-resistant prostate tumor regeneration

**Figure 1:**
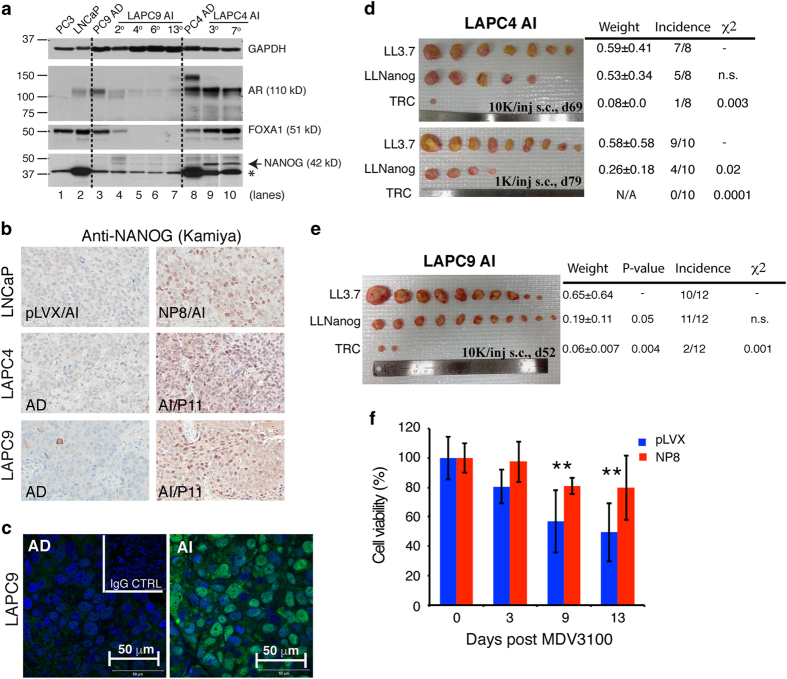
Requirement of NANOG for CRPC growth. (**a**) NANOG western blot analysis (Cell Signaling, D73G4; Supplementary Table S1) in LAPC4 and LAPC9 tumors serially passaged in castrated (AI; passage number indicated) vs intact (AD) hosts. The blot was probed for AR, FOXA1 and GAPDH. *, a non-specific band. Note that the NANOG band upregulated in LAPC9 AI tumors was relatively faint, although the upregulation was corroborated by immunohistochemical (IHC) (**b**) and confocal immunofluorescence (IF) analysis (**c**). (**b**) IHC staining for NANOG (Kamiya; Supplementary Table S1) in AD vs AI LAPC4 and LAPC9 xenografts. Shown on top is the NANOG staining of LNCaP tumors grown in castrated hosts (pLVX, control cells expressing empty vector). (**c**) Representative confocal IF images for NANOG (Cell Signaling, D73G4) in AD vs AI LAPC9 tumors. (**d**, **e**) Freshly purified LAPC4 and LAPC9 AI cells were transduced with the indicated lentiviral Nanog-short interfering RNA construct (vs LL3.7 control) and subcutaneously injected (1 K or 10 K) in castrated nonobese diabetic/severe combined immunodeficiency mice (*n*=8–12). Endpoint tumor weight (mean±s.d.), *P*-values for weight (Student’s *t*-test) and tumor incidence (*χ*^2^-test for statistic) are indicated. (**f**) Enzalutamide resistance in LNCaP cells overexpressing NP8 relative to pLVX control. LNCaP-pLVX and LNCaP-NP8 cells were plated in the presence of DOX (1 μg ml^−1^, 48 h) and then cultured in charcoal-dextran stripped serum plus 40 μm MDV3100 for the indicated time periods. Presented is the % cell viability upon MDV3100 treatment. **P<0.01. NS, nonsignificant.

In PCa, the *NANOG* messenger RNA (mRNA) species are derived, predominantly, from the *NANOGP8* (*NP8*) locus and inducible expression of NP8 imparts castration resistance to LNCaP cells [3, 9]. To address whether endogenous NANOG (note that throughout the text NANOG is frequently used to denote either NP8 or NANOG1) has a causative role in castration-resistant PCa (CRPC), we first generated pairs of androgen-dependent (AD) and AI PCa xenografts, LAPC4 and LAPC9 [11, 13]. Western blot revealed increased 42-kD NANOG protein [14] in both LAPC4 and LAPC9 AI tumors (Figure 1a). Immunohistochemical and immunofluorescence staining using N terminus directed (Kamiya) and C terminus directed (R&D) anti-NANOG antibodies [14] (Supplementary Table S1), validated by NP8-overexpressing LNCaP cells (Figure 1b; Supplementary Figure S1A), corroborated upregulation of NANOG, localized in both nucleus and cytoplasm, in AI xenografts (Figure 1b and c). Notably, the LAPC9 and LAPC4 AI tumors showed opposite patterns of changes in AR and FOXA1 in that the LAPC4 AI tumors showed slight increases in both AR and FOXA1, whereas LAPC9 AI tumors showed decreases in both proteins compared with the corresponding AD tumors (Figure 1a). A pilot immunohistochemical study in a tissue microarray containing 20 CRPC patient samples [15] revealed NANOG-positive cells in ~25% of specimens at various abundance, from undetectable to sporadic positive cells to most cells being positive (Supplementary Figure S1B). As in the AI LAPC9 and LAPC4 xenografts, NANOG in human CRPC samples was detected in the nuclei, nuclear membrane and cytoplasm (Supplementary Figure S1B).

To determine whether the upregulated NANOG in AI tumors is required for CRPC growth, we employed two lentiviral short interfering RNA vectors, that is, LL-Nanog and TRC (targeting 3′-untranslated region and coding region, respectively [3]), to knock down *NANOG* in LAPC4 and LAPC9 AI cells, which were then implanted back into castrated NOD/SCID (nonobese diabetic/severe combined immunodeficiency) mice. As shown in Figure 1d, NANOG knockdown significantly inhibited LAPC4 AI tumor regeneration and the inhibitory effect was particularly strong with TRC vector. The tumor-initiating frequency was reduced from 1/1 654 in the LL3.7 group to 1/6 287 (*P*=0.005) in the LL-Nanog group and to 1/82 900 (*P*=1.33e−08) in the TRC group, respectively (Figure 1d). Similarly, LL-Nanog greatly inhibited tumor growth and TRC short interfering RNA essentially ablated tumor formation (Figure 1e) in LAPC9 AI cells. These results, altogether, suggest that castration *in vivo* upregulates endogenous NANOG in some PCa cells and (clonal) xenografts, and that the upregulated NANOG is functionally required for CRPC maintenance. Consistently, NP8 expression conferred resistance in LNCaP cells to the anti-androgen enzalutamide (MDV3100; Figure 1f).

### Unique pattern of NANOG chromatin occupancy in PCa cells

**Figure 2:**
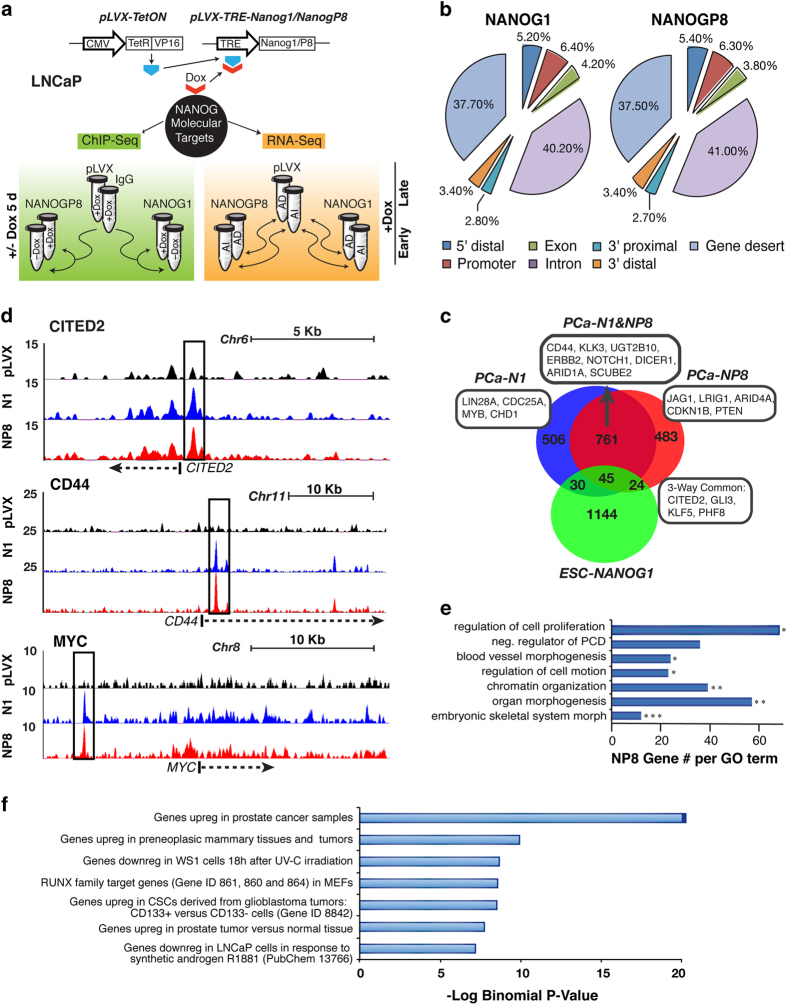
Distinct genomic occupancy of NANOG. (**a**) Scheme for ChIP-Seq and RNA-Seq in LNCaP cells expressing NANOG1 (N1) or NANOGP8 (NP8) vs vector control (pLVX) for the indicated time with or without DOX. (**b**) Genomic distribution of NANOG occupancy relative to the transcription start site (TSS) or transcription end site (TES) of the nearest gene. 5′ distal (−15 kb to −5 kb from the TSS), promoter (−5 kb to +0.5 kb from the TSS), 3′ proximal (−0.5 kb to +5 kb from TES), 3′ distal (+5 kb to +15 kb from the TES); gene desert is all other genomic regions. (**c**) Venn diagram of the promoter region (−8 kb to +2 kb) occupancy of NANOG in ESCs [16] vs N1 and NP8 in LNCaP cells. (**d**) Representative ChIP-Seq traces recovered from the UCSC genome browser. (**e**) Promoter region (−8 kb to +2 kb) occupancy gene ontology (GO) analysis via DAVID. Presented are GO Term Biological Processes, level 4; *P<0.05; **P<0.01; ***P<0.001. (**f**) Distal occupancy GO analysis via GREAT. MSigDB correlations are shown and plotted according to the binomial raw *P*-value.

To understand mechanistically how NANOG reprograms PCa cells to castration resistance [9], we first sought to determine NANOG chromatin occupancy in AD PCa cells. Despite detection of NANOG protein in LAPC4 and LAPC9 AI xenografts, the scarcity of NANOG protein precluded successful immunoprecipitation in multiple attempts. Thus, we took advantage of our established model of initially hormone-dependent LNCaP cells ectopically expressing DOX-inducible NP8 (or NANOG1 for comparison) [9] to perform genome-wide ChIP-Seq and transcriptome (RNA-Seq) analyses (Figure 2a) in a system that recapitulates PCa disease progression via NANOG-mediated reprogramming to castration resistance. ChIP-Seq analysis in LNCaP cells upon 5 days of DOX induction revealed a total of 14 449 NP8 and 14 331 NANOG1 peaks after background subtraction and thresholding for significance (model-basedanalysis of ChIP-Seq, MACS, *P*<e^−5^) (Supplementary Table S2). We classified these NANOG-bound genomic loci to 5′ distal (−15 kb to −5 kb from transcription start site (TSS)), promoter (−5 kb to +0.5 kb from TSS), exon, intron, 3′ proximal (−0.5 kb to +5 kb from transcription termination site), 3′ distal (+5 kb to +15 kb from transcription termination site) and gene desert (Figure 2b). The genomic occupancy profile for NP8 was overall similar to that of NANOG1 (Figure 2b).

On the other hand, genomic occupancy of NANOG in LNCaP cells differed from that of NANOG1 in ESCs [16] (Figure 2c), suggesting significant differences between NANOG in ESCs and in cancer cells. Using a definition of extended ‘promoter region’ (that is, −8 kb to +2 kb from TSS [16]), we observed 1 313 high-fidelity promoter region binding sites for NP8 and 1 342 sites for NANOG1 in LNCaP cells, ~60% (806 sites) of which were shared (Figure 2c). Examples of three-way commonly occupied genes included *KLF5*, *CITED2*, *GLI3* and *PHF8*, whereas ‘NP8-only’ ChIP peaks occurred in the promoters of genes such as *JAG1*, *LRIG1*, *WNT5A*, *ARID4A/4B*, *HIF1A*, *SMAD6*, *PTEN* and *PSCA* (Figure 2c; representative tracks shown in Figure 2d; Supplementary Figure S2A and B). Notably, a close examination of ChIP-Seq signals revealed that the majority of NP8-only or NANOG1-only loci (Figure 2c) were also occupied by the other NANOG counterpart, as evident from the examples shown (*LRIG1* and *JAG1*; Supplementary Figure S2B). Thus, NP8 (and NANOG1) occupancy of the LNCaP cell genome is typified by preferential rather than distinct binding. Among the 806 NANOG1 and NP8 commonly occupied promoters in LNCaP cells were many developmentally related genes including *HOXA3* and *A4*; *HOXC4* and *C9*; *LIN7A*, *7C* and *37*; *DICER1*, *NOTCH1*, *HES6*, *GLI3* and *TBX3*; chromatin remodeling and epigenetic regulators important for SC functions such as *KDM4A*, *4B* and *4D*; *CITED2*, *PHF8*, *ARID1A* and *ARID5A*; *TRIM68* and *PRMT3/8*; and molecules implicated in CSCs (for example, *CD44* and *KLF5*) and PCa development (for example, *TPD52* ; Figure 2c and d; Supplementary Figure S2A and B).

No direct binding of NP8 (or NANOG1) to *AR* promoter regions was found (Supplementary Table S2) despite lower levels of AR in NANOG-overexpressing PCa cells or tumors [9]. Interestingly, NANOG peaks were also detected ~10 kb upstream from the TSS for two genes previously shown to be transcriptionally upregulated by NANOG in PCa cells [9], namely, *c-MYC* (Figure 2d) and *ABCG2* (not shown). Gene ontology (GO) analysis of promoter region (−8 kb to +2 kb) occupancy using DAVID classified the promoter binding of NP8 in LNCaP cells into various functional categories, such as regulation of cell proliferation (GO: 0042127), negative regulation of cell death (GO: 0043069) and regulation of cell motion (GO: 0051270; Figure 2e). GO analysis of distal chromatin (defined by the −8 kb to +2 kb promoter region plus a 200 kb extension in both directions) occupancy by GREAT (Gene Region Enrichment and Annotation Tool [17]) revealed an association with PCa, CSC traits and steroid hormone receptor signaling (Figure 2f) including AR-responsive (both up- and downregulated) genes such as *KLK3*, *CDK1*, *NKX3.1*, *TPD52*, *TMPRSS2*, *LRIG1* and *SOX9* (Supplementary Figure S2C).

### NANOG occupancy in PCa cells converges on FOXA1 and AR signaling

**Figure 3:**
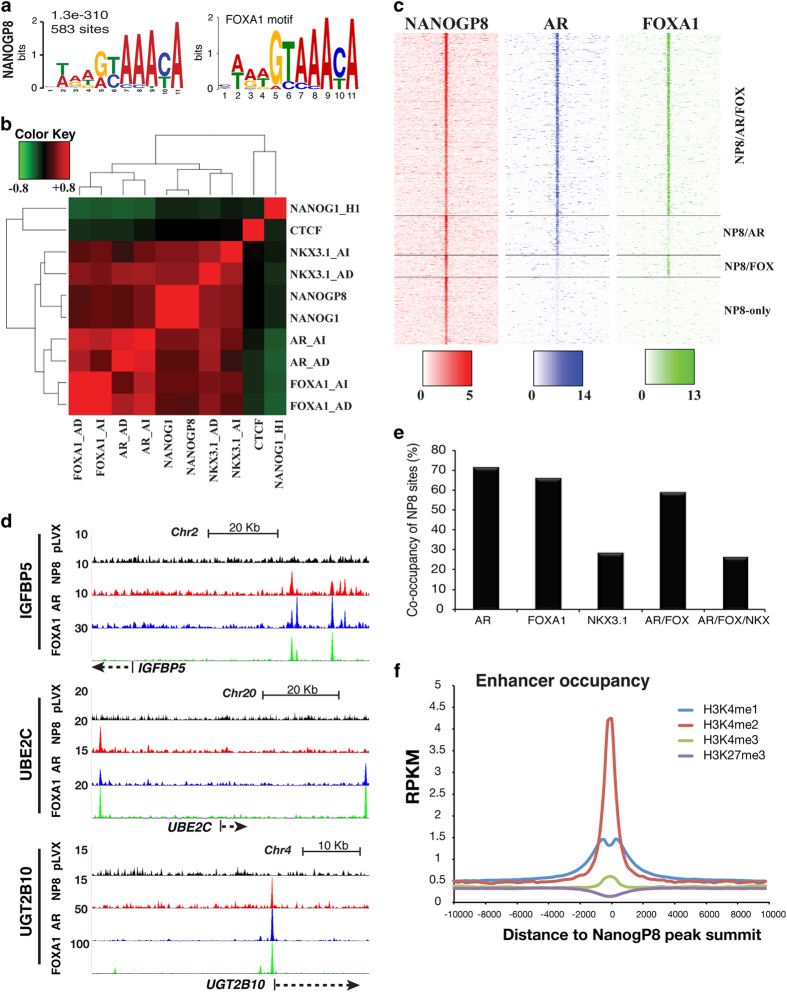
NANOG co-occupies the AR and FOXA1 sites in LNCaP cell genome. (**a**) MEME motif analysis identifies the FOXA1 motif (right) as the most frequently occupied motif by NP8 (the E-value for the occurrence of this motif=1.3e−310) in LNCaP cells. (**b**) Pearson’s correlation of transcription factor (NANOG, AR, FOXA1 and NKX3.1) chromatin occupancy in LNCaP under AD and AI conditions. CTCF occupancy in LNCaP and NANOG1 occupancy in H1 ESCs are shown for comparison. (**c**) Signal distribution heatmap analysis of peaks (NANOGP8, FOXA1 and AR) centered on NANOGP8, ±10 kb from the peak, sorted according to NANOG peak intensity and grouped according to classification (three-way common, two-way common and NP8 only) as indicated. (**d**) Representative ChIP-Seq tracings reveal multiple transcription factor loci co-occupied by NANOG. (**e**) Bar chart showing the proportion of NANOG-binding sites co-occupied by FOXA1, AR and/or NKX3.1 in the presence of androgen. (**f**) Distribution of histone marks ±10 kb around NP8 ChIP-Seq peaks occurring in non-promoter occupied regions (peaks excluded from −8 kb to +2 kb relative to a TSS). H3K4me1, H3K4me3 and H3K27me3 data were acquired by ChIP-Seq; H3K4me2 data are from published data (GSM503905). RPKM, reads per kilobase of transcript per million mapped reads.

We next performed MEME motif analysis [18] of NANOG-occupied chromatin in LNCaP cells using the top 800 peaks ±100 bp of the pinnacle. Surprisingly, the most significantly enriched motif in NP8 occupied chromatin was that of FOXA1 (583 sites/800 peaks, Figure 3a; the overlap between the two motifs is highly statistically significant with *P*=4.83e−11), a pioneering TF for steroid hormone receptor signaling and a known AR-interacting protein [19–21]. A similar finding was made for NANOG1 (689 sites/800 peaks, *P*=8.12e−06; Supplementary Figure S3A). This observation suggested that NANOG occupies regions of the chromatin regulated by AR signaling, and that NANOG reprograms PCa cells by converging on steroid hormone receptor signaling. The second most common motif (138 sites/800 peaks; *P*=1.56e−04) occupied by NP8 in LNCaP cells was related to the consensus motif (C(T/C)TGGC(A/T)) of the nuclear factor I (NFI) family, recently shown to be another FOXA1/AR complex factor [22], further strengthening the interactive relationship between NANOG and steroid hormone receptor signaling components.

A meta-analysis comparing NP8 chromatin occupancy with that of steroid receptor complex proteins AR, FOXA1 and NKX3.1 revealed coordinate occupancy of these factors in both AD and AI conditions (Figure 3b). Pearson’s correlation confirmed concordant genome-wide NANOG1 and NP8 occupancy, and further showed that NANOG occupancy in PCa cells was distinct from either CTCF occupancy in PCa cells or NANOG1 occupancy in H1 ESCs (Figure 3b). Critically, these comparisons also indicated that NANOG occupancy under AD conditions more closely resembled AR occupancy in AI conditions (Figure 3b), suggesting that NANOG may ‘poise’ PCa cells for reprogramming to castration resistance. Center of distribution heatmap analysis of peaks (NP8, FOXA1 and AR) centered on NP8 (±10 kb from the peak) confirmed that NANOG occupancy overlapped with that of AR and FOXA1, and NKX3.1 (Figure 3c and d; Supplementary Figures S2C and D, and S3B). Notably, detection of NP8-only peaks (3 058, 21%; Figure 3c; Supplementary Figure S3B) suggests that NANOG occupancy is not absolutely contingent upon the presence of other steroid hormone receptor complex proteins.

Quantification of the 14 449 NANOGP8 peaks that overlapped with sites occupied by FOXA1, AR and/or NKX3.1 in the presence (Figure 3e) or absence (Supplementary Figure S3C) of androgen demonstrated that AR-binding sites encompassed the largest proportion (~71%) of NANOG-occupied regions under AD conditions (Figure 3e), whereas FOXA1-binding sites encompassed the largest proportion (~71%) of NANOG-occupied regions under AI conditions (Supplementary Figure S3C). Nevertheless, >50% of NANOG-occupied chromatin overlapped with sites co-occupied by both AR and FOXA1 in both the presence and absence of androgen (Figure 3e; Supplementary Figure S3C). Of the three proteins subjected to meta-analysis, NKX3.1-binding sites represented the smallest fraction (~25%) of NANOG-occupied genomic regions (Figure 3e; Supplementary Figure S3C). An analysis from the opposite perspective revealed that only a modest proportion of total AR or FOXA1 occupied loci (in the presence or absence of androgen) overlapped with NP8-binding sites (Supplementary Figure S3D). Interestingly, however, a greater percentage (~40%) of the less frequent NKX3.1-occupied regions (relative to the more abundant AR and FOXA1 sites) were bound by NP8 (Supplementary Figure S3D).

We performed a comparison of NP8 genomic occupancy in LNCaP cells with baseline histone-methyl marks and observed that NANOG promoter occupancy corresponded with H3K4me2 and H3K4me3, but not H3K27me3 (Supplementary Figure S3E), suggesting that NANOG typically occupies initially transcriptionally active promoters. At distal regions, NANOG was predominantly enriched for H3K4me2, followed by H3K4me1, but not H3K27me3 (Figure 3f), implying that NANOG preferentially occupies transcriptionally active enhancer regions.

### NP8 directly interacts with AR and FOXA1

**Figure 4:**
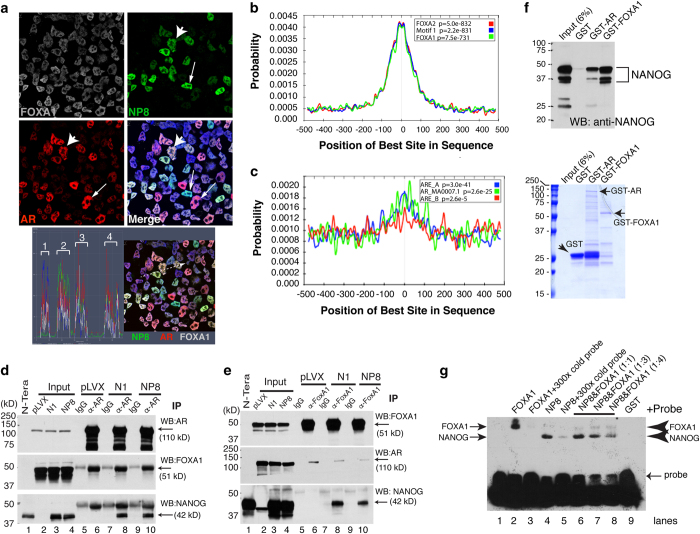
NANOG co-localizes and interacts with AR and FOXA1. (**a**) Multispectral confocal immunofluorescence analysis of NANOG (rabbit mAb, green), AR (mouse mAb, red) and FOXA1 (goat pAb, gray) in LNCaP-NP8 cells (the lower right panel being the four-color merge). Cells were counterstained by 4,6-diamidino-2-phenylindole (blue). Arrows indicate NP8- or AR-expressing cells, whereas the arrowhead marks a NP8/AR co-expressing cell. Shown below are semi-quantitative spectral peaks of four cells (left) circumscribed on the right image in white dashed line. (**b**) CentriMo analysis of the positional distribution of FOXA family motifs (motif 1, FOXA1 and FOXA2) ±500 bp of the pinnacle. (**c**) CentriMo analysis of the positional distribution of AR family motifs (AR full motif MA0007.1 and AR half motifs ARE_A and ARE_B) ±500 bp of the pinnacle. (**d**, **e**) NANOG interacts with both AR and FOXA1 in LNCaP cells. Whole-cell lysate from the indicated cell types (that is, LNCaP-pLVX control and LNCaP-overexpressing NANOG1 or NP8) were used in immunoprecipitation (IP) with either anti-AR rabbit pAb followed by western blot (WB) with anti-AR mouse mAb, anti-FOXA1 goat pAb and anti-NANOG mouse mAb (**d**) or IP with anti-FOXA1 goat pAb followed by WB with anti-FOXA1 rabbit pAb, anti-AR mouse mAb and anti-NANOG rabbit mAb (**e**). N-tera EC cell lysate was used as a positive control for NANOG (42 kDa; lane 1). (**f**) Recombinant NP8 interacts with both AR and FOXA1 in cell-free systems. GST pull-down assays were performed as described in Supplementary Methods and bound proteins were separated by SDS–polyacrylamide gel electrophoresis (SDS/PAGE) and used in WB with an anti-NANOG antibody (Cell Signaling). Shown below is a Coomassie blue-stained gel image. The rhNANOG proteins were detected as 48, 42 and 35 kDa species [14]. (**g**) NP8 binds the FOXA1 consensus DNA motif. EMSA was performed using biotinylated FOXA1 motif in the *UBE2C* gene promoter as the probe (lane 1). Upon incubating the indicated recombinant proteins (lanes 2–8) or GST alone (lane 9) with the probe, interacting proteins were separated by SDS–PAGE and detected using streptavidin horseradish peroxidase. Note that cold unlabeled probes significantly reduced binding of FOXA1 (lane 3) or NP8 (lane 5) to the biotinylated probe. The arrowheads (right) indicate the increasing amounts of FOXA1 and decreasing amounts of NANOG with increasing ratio of FOXA1 over NP8 (lanes 6–8).

Confocal immunofluorescence microcopy staining for NP8, FOXA1 and AR revealed FOXA1 to be the most homogeneously (and abundantly) expressed of the factors, with AR^+/hi^ and NP8^+/hi^ cells heterogeneously observed (Figure 4a). In a subset of cells, NP8 and AR were co-expressed (arrowheads), whereas in other cells, an inverse correlation was apparent (Figure 4a; arrows). Nevertheless, the majority of the cells expressed detectable NP8 and AR (Figure 4a; profile analysis shown below). Thus, NANOG, AR and FOXA1 are frequently co-localized to the nucleus in a subset of PCa cells with the potential to directly interact.

Conceptually, the impinging of NANOG on AR signaling could be via its interaction with FOXA1, AR or other factors known to interact with AR (for example, LSD1 and NKX3.1) [23, 24]. Centralized localization of FOXA1 (or AR) motifs with NANOG-binding loci may indicate direct protein–protein interactions and coordinated regulation of gene expression programs. MEME and CentriMo analysis of 600 randomly selected peaks revealed that motif 1 (630 sites; *E*=3.1e−272) of the FOXA family proteins was centrally distributed in the majority of NANOGP8 peaks (*P*=2.2e−831; Figure 4b). Such analysis also identified a second relatively common motif (165 sites; *E*=1.1e−60), corresponding to either the half or full NFI family consensus motif TTGGCN5GCCAA, that was also centrally distributed but to a lesser degree than FOXA1 (*P*=4e^−118^; Supplementary Figure S4A). Finally, although the canonical AR full motif (MA0007.1) was scarce among NANOG peaks, an evaluation of AR half motifs (ARE_A and ARE_B) revealed that ~10% (722/6 450) of the NP8 peaks harbored a detectable AR-binding site (*P*<0.001) that was only modestly centralized (Figure 4c; *P*=3e^−41^). These *in silico* analyses further support that NANOG may directly interact with FOXA1 and/or AR.

To directly interrogate potential NANOG and FOXA1/AR protein interactions, we first performed proximity ligation assays (PLA) to determine whether the proteins were in close spatial proximity (that is, within ~40 nm). We detected, as expected, strong interaction signals between AR and FOXA1 (Supplementary Figure S4B). We also detected positive NANOG and AR PLA signals in most NANOG1- and NP8-expressing LNCaP cells (Supplementary Figure S4B). Surprisingly, however, PLA signals were not observed with anti-NANOG and anti-FOXA1 staining (data not shown), suggesting that either the two molecules (that is, NANOG and FOXA1) are not within the 40 nm range or the two antibodies used are not compatible for the PLA. We subsequently performed co-immunoprecipitation (co-IP) analysis, which showed that AR co-IP pulled down AR and FOXA1 in all groups (pLVX, NANOG1 and NP8) but pulled down NANOG only in NANOG1/NP8-expressing LNCaP cells (Figure 4d). Similarly, anti-FOXA1 immunoprecipitation pulled down AR in all three groups but co-IP’ed down NANOG exclusively in NANOG1/NANOGP8-expressing cells (Figure 4e). These results suggest that NP8 (and NANOG1) may directly interact with AR or FOXA1. In support, glutathione *S*-transferase (GST) pull-down assays revealed that both GST-AR and GST-FOXA1 fusion proteins pulled down the 42 and 48 kDa [14] recombinant human NP8 proteins (Figure 4f). Finally, electrophoretic mobility shift assay (EMSA) assays using the FOXA1 motif in the *UBE2C* gene promoter as the probe demonstrated that recombinant NP8 as well as the FOXA1 control could bind to biotinylated FOXA1 motif, and this binding was competed out by 300× cold probe (Figure 4g; Supplementary Figure S4C and D). Notably, at a 1:1 ratio NP8 demonstrated stronger binding to the FOXA1 motif than FOXA1 (Figure 4g; Supplementary Figure S4C and D), although the NP8 binding was slightly attenuated by increasing amounts of FOXA1 (Figure 4g; Supplementary Figure S4E). Taken together, these data suggest that NP8 can directly interact with AR and FOXA1, and directly bind the FOXA1 genomic motif.

### NP8 both represses and activates distinct clusters of AR-regulated genes: association with castration resistance and correlation with patient outcomes

**Figure 5:**
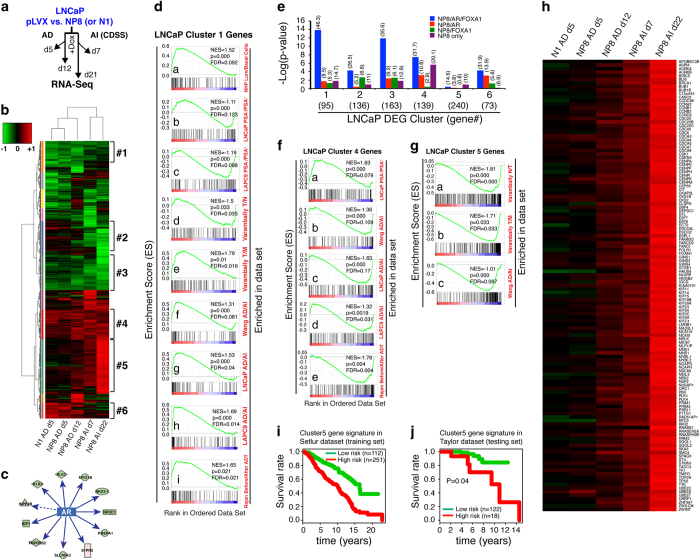
NANOG induces distinct gene expression changes correlated with castration resistance and patient survival. (**a**) Schematic showing RNA-Seq analysis of LNCaP cells overexpressing NANOG in either androgen-dependent (AD) or in androgen-independent (AI) conditions for the indicated time. (**b**) Unsupervised hierarchical clustering and heatmap presentation of all DEGs from the indicated groups (up/down >1.5× and *P*<0.05 relative to the pLVX control) in at least one group. Shown on the right are six clusters of genes that showed distinct patterns (Supplementary Figure S5). (**c**) IPA Upstream Regulator analysis implicated AR as a key NANOG target gene repressed under AD d5 (activation *Z*-score=−2.9), manifested by the downregulation of multiple AR target genes. (**d**) GSEA of cluster 1 genes in multiple data sets link them as AR-regulated pro-differentiation genes. (**e**) Integrative analysis of NP8 genomic occupancy (ChIP-Seq) and NANOG-induced DEG clusters. The analysis was performed by Fisher’s exact test to determine the enrichment of DEGs co-occupied by NP8 with AR and/or FOXA1 within a ±50 kb window of each peak. (**f**, **g**) GSEA of cluster 4 (**f**) and cluster 5 (**g**) genes in the data sets indicated implicate their involvement in castration resistance. (**h**) Heatmap presentation of the 127 genes in cluster 5 involved in DNA replication, cell cycle regulation and cytokinesis. (**i**, **j**) Survival analysis links cluster 5 genes to poor patient survival. A 58-gene signature from cluster 5 genes was used to stratify PCa patient survival in the Setlur data set (Supplementary Method), in which patients with higher expression of the signature had significantly shorter overall survival (that is, high risk of dying) than those with lower expression of the signature (**i**). The same signature also predicts for poor patient survival in a testing data set (**j**). In cluster 5, higher expression corresponds to higher risk.

We performed genome-wide RNA-Seq analysis under both AD and AI conditions in NP8-expressing LNCaP cells both short-term (d5 and d7 for AD and AI, respectively) and long-term (d12 and d22 for AD and AI, respectively) relative to pLVX (Figure 2a and 5a). As comparison, we also performed RNA-Seq in NANOG1 (N1) d5 cells under AD. Unsupervised clustering of differentially expressed genes (DEGs; up/down>1.5 and *P*<0.05; Figure 5b; Supplementary Tables S3–S6; Supplementary Figure S5) revealed the following important points: (1) N1 and NP8 elicited similar overall transcriptional responses; (2) ‘clusters’ of DEGs with distinct patterns of changes were observed; (3) some DEGs were consistent across all conditions and insensitive to the time course (short-term vs long-term) or androgen environment (for example, gene clusters #1, #3 and #4; see below); and (4) other DEGs showed time- and androgen status-dependent changes (for example, gene clusters #2, #5 and #6).

The 95 genes in cluster 1, which were persistently repressed by NANOG under both AD and AI conditions (Figure 5b), contained many ‘conventional’ AR downstream targets normally involved in differentiation including *KLK3, KLK2*, *NKX3.1, TMPRSS2*, *RDH11, STEAP1, PEG3, SPDEF, LRIG1, TRPM8, ELL2* and *ACSL3* (Supplementary Figure S5). Ingenuity pathway analysis (IPA) ‘Upstream Regulator’ analysis of all DEGs in AD d5 cells also revealed NANOG-mediated AR inhibition manifested as downregulation of classic AR targets such as *KLK3*, *NKX3.1* and *IGF1* (Figure 5c; *P*=2.28e−4; *Z*-score=−2.9). A similar AR inhibitory effect was observed in NP8 AD d12 cells (*P*=3.53e−5; Z-score=−2.2). Comprehensive Gene Set Enrichment Analysis (GSEA; Supplementary Method) revealed that the cluster 1 genes were enriched in normal human prostate differentiated (AR^+^/PSA^+^) luminal cells, PSA^+^ PCa cells, primary adenocarcinomas, AD PCa cell lines and AD xenograft tumors, and, importantly, patient primary tumors before androgen deprivation therapy (ADT) (Figure 5d, a-i). These results suggest that the cluster 1 genes are associated with AR-regulated cellular differentiation and with sensitivity to ADT. Interestingly, cluster 3 genes (163) showed a very similar NANOG-repressed pattern as cluster 1 genes (Figure 5b), suggesting that these genes might also be AR-regulated, differentiation-related genes repressed by NANOG. Indeed, GSEA revealed an enrichment pattern of cluster 3 genes similar to the cluster 1 genes (Supplementary Figure S6A). Strikingly, GSEA of the 258 genes in clusters 1 and 3 revealed the enrichment of these genes in the same cohort of data sets (Supplementary Figure S6B). Importantly, the Upstream Regulator analysis of the 258 genes revealed numerous genes as downstream targets of androgens and R1881 (Supplementary Figure S6C). Integrative analysis of our ChIP-Seq and RNA-Seq data demonstrated that genes in clusters 1 and 3 were most highly enriched in co-occupancy by NP8/AR/FOXA1 (Figure 5e; Supplementary Figure S6D). These results suggest that NANOG, under both AD and AI conditions, persistently inhibits a set of androgen/AR-regulated pro-differentiation genes potentially mediating castration resistance and influencing patient outcome. In support, Oncomine concept analysis showed that 42 of the 258 (16%) genes in clusters 1 and 3 were under-expressed in PCa compared with normal/benign tissues and 58 of the 258 (22.5%) genes were downregulated in PCa metastases compared with primary tumors (Supplementary Figure S6E and F; data not shown). We derived a 33-gene signature from the cluster 1/3 genes and found that this signature not only was able to stratify PCa patients into groups of low vs high risk of dying but also could predict for favorable patient survival in an independent cohort (Supplementary Figure S6G and H).

Genes in clusters 2 (136) and 6 (73) followed somewhat a similar pattern of changes: upregulated at AD d5, slightly downregulated at AD d12 and then significantly downregulated in AI conditions (Figure 5b). These genes could theoretically be rapidly induced by NANOG to initiate the CRPC reprogramming, as supported by GSEA showing both clusters enriched in patients’ CRPC tumors (Supplementary Figure S6I and J). These two clusters of genes showed less enrichment, compared with the clusters 1 and 3 genes, in NP8/AR/FOXA1 co-occupancy (Figure 5e; Supplementary Figure S6D), although ~40% and 50%, respectively, of the peaks were co-occupied by NP8 with AR and/or FOXA1 (Figure 5e). These results imply that perhaps as many as half of the genes in these two clusters were activated early (that is, at d5) by NP8 together with AR and/or FOXA1, and their downregulation in AI conditions (Figure 5b) further suggests that ligand-activated AR signaling is required for their sustained regulation by NANOG.

The 139 genes in cluster 4, in contrast to those in clusters 1 and 3, were persistently activated by NANOG under both AD and AI conditions (Figure 5b). GSEA showed enrichment of the cluster 4 genes in LAPC9 AI cells as well as in patients’ post-ADT CRPC tumors (Figure 5f), suggesting that the upregulation of these genes was associated with castration resistance. Notably, this cluster included, in addition to transgene *NANOG*, many SC genes such as *LMO4, LMO2, TERT, HOXD8* and *SOX7* (Supplementary Figure S5). Integrative analysis of ChIP-Seq and the DEGs revealed, interestingly, a relative enrichment in cluster 4 genes for genomic binding by NP8 only (that is, in the absence of AR and/or FOXA1; Figure 5e; Supplementary Figure S6D). Motif analysis revealed that of the 3 128 total NP8-only peaks the most commons motifs were FOX family-like (for example, FOXC1) and NFI family binding motifs (data not shown).

### NP8 time-dependent cell cycle gene induction correlates with poor patient survival

Strikingly, the 240 genes in cluster 5 showed time-dependent upregulation and further upregulation under AI conditions (Figure 5b; Supplementary Figure S5), suggesting that these genes are gradually activated by NANOG and further upregulated, synergistically, by NANOG and castration and might also be involved in castration resistance and associated with PCa aggressiveness (Figure 5g). GSEA of the NP8 AI d22 DEGs revealed a great enrichment in cell cycle and DNA replication categories (Supplementary Figure S6K). Remarkably, knowledge-based annotation of each gene [11, 25] demonstrated that >50% of the cluster 5 genes were involved in DNA replication, cell cycle progression and mitosis (spindle formation and checkpoint, cytokinesis, and so on; Figure 5h). Notable examples included *AURKA/B*, *BUB1, FOXM1, PLK1/4, UBE2C/2S/2T*, and multiple *cyclin*, *CDK, CDC, CENP* and *KIF* genes (Figure 5h). Many of these ‘M-phase’ genes such as *UBE2C*, *FOXM1*, *CDK1* and *CDC20* have been previously linked to PCa progression [26] and AR-mediated castration resistance under castrated conditions [20]. In support, GSEA demonstrated that the cluster 5 genes were greatly enriched in prostate tumors (in comparison with normal tissues), metastases (over primary tumors), and AI tumors (in comparison with AD tumors; Figure 5g). Furthermore, Oncomine concept analysis showed that 59 of the 240 (25%) genes in cluster 5 were upregulated in PCa compared with normal tissues and, remarkably, 142/240 (59%) genes were overexpressed in PCa metastases compared with primary tumors (Supplementary Figure S6L; data not shown), strongly linking this cluster of genes to PCa aggressiveness. We derived a 58-gene signature from the cluster 5 genes, which was found to be associated with and also predictive of worse patient OS (Figure 5i and j; data not shown).

Included in cluster 5 were also many genes involved in cell migration/motility (11%), epigenetics (~7%; including many histone 1 genes) and in neurogenesis and neuronal development such as *CTSL2, MPP6, CADPS2*, *NXNL2, NEUL1B* and *ADORA1* (Supplementary Figure S5). Surprisingly, integrative ChIP-Seq and RNA-Seq analysis revealed that the cluster 5 DEGs were not significantly enriched in genomic binding by NP8, AR or FOXA1 (Figure 5e; Supplementary Figure S6D), suggesting that the majority of these genes are indirect NANOG targets.

### NP8 upregulates cell motility genes and engages the MYC transcriptional program

**Figure 6:**
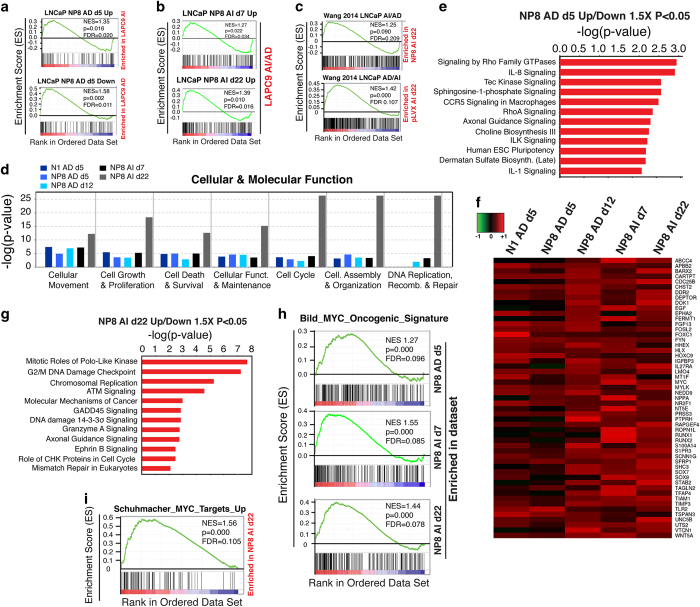
NANOG upregulates cell motility genes and engages oncogenic MYC. (**a**–**c**) GSEA showing that NP8 upregulated genes at AD d5 (**a**, top) or AI d7 and d22 (**b**) were all enriched in LAPC9 AI tumors. In contrast, NP8 downregulated genes at AD d5 were enriched in LAPC9 AD tumors (**a**, bottom). Reciprocally, the LNCaP AI-Up genes were enriched in the NP8 AI d22 cells, whereas LNCaP AD-Up genes were enriched in the control (pLVX) AI d22 cells (**c**). (**d**) IPA Biological Processes analysis of NANOG-induced DEGs (both up/down >1.5× and *P*<0.05) under indicated five conditions. Presented are the major ‘Cellular & Molecular Functions’ enriched in each condition. (**e**) IPA Canonical Pathway analysis of DEGs (both up/down >1.5× and *P*<0.05) under AD d5 in response to NP8 overexpression. (**f**) Heatmap of the 53 migration-related genes (from Supplementary Figure S7G; presented here alphabetically) persistently induced by NANOG expression. (**g**) IPA Canonical Pathway analysis of DEGs (both up/down >1.5× and *P*<0.05) in AI d22 cells. (**h**, **i**) GSEA showing the MYC oncogenic signature (**h**) and MYC target genes (**i**) are enriched in both NP8 AD and AI LNCaP cells.

We next sought to dissect the context-dependent and dynamic changes in global gene expression by analyzing the distinct DEG profiles under each condition. We found that NP8 upregulated genes (>1.5×, *P*<0.05), at AD d5 or AD d12, or at AI d7 or d22, were associated with castration resistance (Figure 6a–c; data not shown). IPA Biological Processes analysis of NANOG-induced up- and downregulated DEGs (>1.5× and <0.67, *P*<0.05; Supplementary Tables S3–S6) implicated gene expression changes in ‘Cellular Movement’, ‘Cell Growth & Proliferation’ and ‘Cell Survival’ (Figure 6d). Consistent with our demonstration that one of the earliest and most significant changes elicited by NANOG is the increased cell motility [9], many biological pathways regulating cell migration including Rho GTPase signaling and integrin-linked kinase signaling were altered in NP8 AD d5 cells (Figure 6e). Furthermore, ‘Cellular Movement’ was ranked the top category among AD d5 (and d12) and AI d7 upregulated genes (Supplementary Figure S7A–D). In fact, the number of cell motility-related genes continued to increase in a time-dependent manner and during AD to AI transition (Supplementary Figure S7E and F). Although some migration/metastasis related genes including *IGFBP5* and *CXCR4* that we previously reported to be induced by NANOG [9] were gradually upregulated (Supplementary Figure S7G, arrows), ~50 motility genes were persistently induced (Figure 6f). Notably, however, under the concerted actions of NANOG and castration and with time, genes associated with ‘Cell Cycle’, ‘Cell Growth & Proliferation’ and ‘DNA Replication & Repair’ became the predominant categories in NP8 AI d22 cells (Supplementary Figure S7A–D), consistent with canonical pathway analysis (Figure 6g) and our prior manual annotation (Figure 5h). Indeed, among the top ranked gene subcategories were ‘M-phase’ (*P*=6.2e^−29^; activation *Z*-score=2.102) and ‘Cell Growth & Proliferation’ (*P*=1.0e^−13^; activation *Z*-score=6.635).

Consistent with a time-dependent induction by NANOG of MYC mRNA (Figure 5h) and protein [9] (Supplementary Figure S7H, data not shown), IPA Upstream Regulator analysis implicated MYC as a key NANOG downstream target (activation *Z*-score=4.262; *P*=6e^−7^; Supplementary Figure S7I). The NP8 upregulated DEGs, under both AD and AI conditions, were highly enriched in MYC signatures or targets (Figure 6h and i). Interestingly, many MYC responsive NP8 AI d22 DEGs overlapped with the targets of FOXM1 (Supplementary Figure S7I), a known cell cycle regulator also induced by NANOG (Figure 5h). These observations thus implicated FOXM1 as a potential downstream mediator of NP8 functions (activation *Z*-score=4.578; *P*=1e^−23^). Also activated was CCND1 (activation *Z*-score=4.536; *P*=1e^−48^), whereas the cyclin-dependent kinase inhibitor CDKN1 (p21) was strongly repressed (activation *Z*-score=−4.158; *P*=1e^−53^). As MYC and FOXM1 have critical roles in both cell cycle progression and motility [26], these analyses suggest that NANOG-mediated PCa cell reprogramming may likely intersect with and engage both transcriptional programs.

### Biological integration of NANOG-reprogrammed PCa cell resistance to androgen deprivation

**Figure 7:**
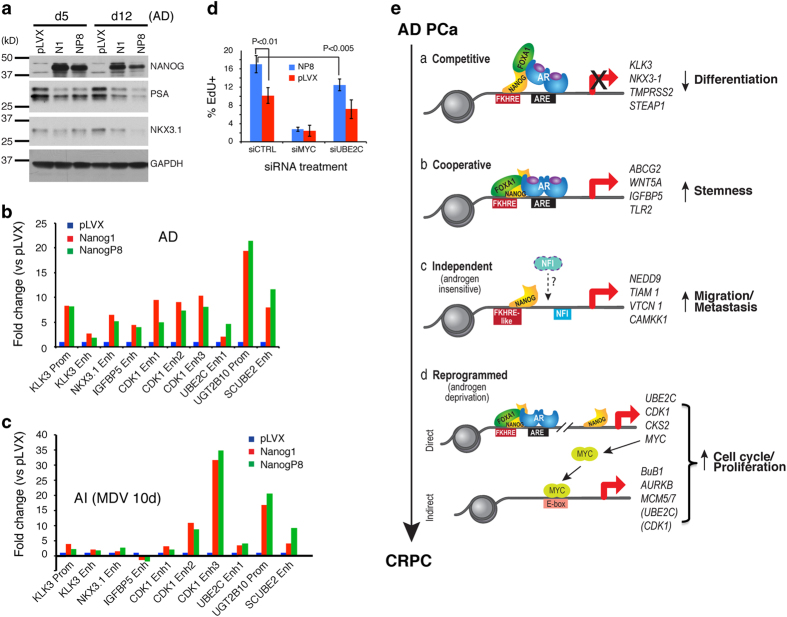
Biological integration of NANOG-reprogrammed PCa cell resistance to androgen deprivation. (**a**) WB analysis of two AR-regulated differentiation proteins (PSA and NKX3.1) in LNCaP cells expressing NANOG1 (N1) or NANOGP8 (NP8) under regular culture (AD) conditions. (**b**, **c**) ChIP–qPCR analysis of NANOG binding to the promoter (Prom) and/or enhancers (Enh) of the indicated genes (see Supplementary Figure S8A for locations of the loci) in Dox-induced LNCaP cells under AD (**b**) and AI (**c**) conditions. ChIP was performed with R&D anti-NANOG goat pAb. (**d**) Functional analysis of NANOG target genes by siRNA-mediated knockdown. NP8-expressing and control (pLVX) LNCaP cells maintained in charcoal-dextran stripped serum and transfected with the siRNAs (100 nm, 72 h) against UBE2C or MYC (positive control) were used in cell proliferation assays. Presented are % EdU^+^ cells (mean±s.e.m.; *n*=3). (**e**) A model depicting modes of operation (a–d) of NANOG during reprogramming of androgen-dependent PCa cells to the CRPC state.

The above integrative ChIP-Seq and RNA-Seq analysis indicates that NANOG persistently represses several hundred AR-regulated pro-differentiation genes, as supported by decreased protein levels of PSA and NKX3.1 (Figure 7a). ChIP–quantitative PCR (qPCR) analysis in fresh samples against select peaks (Supplementary Figure S8A) identified by ChIP-Seq and using primers targeting these loci (Supplementary Table S7) revealed that, consistent with the ChIP-Seq data (Supplementary Figure S2C), both NANOG1 and NP8 occupied the promoter and enhancer regions of *KLK3* gene and an enhancer in *NKX3.1* gene under AD conditions (Figure 7b), and the occupancy was reduced in AI cells (that is, cells treated with 20 μm MDV3100 for 10 days; Figure 7c). Also, consistent with ChIP-Seq data (Figure 3d), NANOG bound to an enhancer of *IGFBP5* (Figure 7b), a known AR target gene, in LNCaP AD cells but the binding was reduced in AI cells (Figure 7c).

RNA-Seq analysis also revealed induction by NANOG of multiple cell cycle genes, especially under AI conditions (Figure 5h). Consistent with this gene expression profile, NP8-expressing cells chronically exposed to MDV3100 (20 μm; 30 days) proliferated faster than control cells (Supplementary Figure S8B; 37.0±0.4% EdU^+^ cells in NP8-expressing cultures vs 16.5±12.4% EdU^+^ cells in control; *P*=0.02, *n*=3). Interestingly, ChIP–qPCR analysis of *CDK1* genomic binding (Supplementary Figure S8A; Supplementary Figure S2C) detected NANOG binding, nearly equally, to the three enhancers of the *CDK1* gene in AD cells (Figure 7b) but, strikingly, NANOG binding to enhancer 3 was greatly increased in AI cells (Figure 7c). This shift in a specific enhancer binding might underlie increased *CDK1* mRNA expression (Figure 5h) and cell proliferation (Figure 7d) under AI conditions. On the other hand, NANOG binding to the enhancer region of *UBE2C* (Figure 3d; Supplementary Figure S8A), another cell cycle regulator implicated in PCa cell castration resistance [20], did not change in AD vs AI LNCaP cells (Figure 7b and c), although the UBE2C protein significantly increased in long-term castrated NANOG-expressing cells (Supplementary Figure S8C) similar to the increase in its mRNA levels under AI conditions (Figure 5h). Importantly, the increased UBE2C mRNA/protein levels were biologically relevant as the siRNA-mediated UBE2C knockdown reduced proliferation in NP8-expressing cells cultured in AI conditions (Figure 7d). In these experiments, we also noted that NP8-expressing LNCaP cells proliferated faster than the control (pLVX) cells (Figure 7d; the siCTRL bars), and that siMYC caused significant inhibition of proliferation in both NP8-expressing and control cells (Figure 7d), implicating the general importance of MYC in the proliferation of LNCaP cells. Similar to UBE2C, other two molecules, that is, UGT2B10 (Figure 3d) and SCUBE2 (Supplementary Figure S2D), that we interrogated by qPCR also did not show marked changes in NANOG occupancy in AI (Figure 7c) compared with AD cells (Figure 7b).

## Discussion

Strong experimental and correlative clinical evidence suggests that NANOG (NANOG or NP8) is expressed in subpopulations of cancer cells and has functional roles in mediating CSC properties, cell motility and invasion, therapy resistance, and metastasis [1, 3,5,6,7,8,9,27, 28,29]. A causal role of NANOG in tumorigenesis has been demonstrated not only by loss- and gain-of-function studies in cancer cells [1, 3, 9] but also by, recently, CRISPR/Cas9-mediated knockout [31] and genetic mouse model [28, 32, 33,34] studies. In addition to constitutive expression of NANOG in a small subset of cancer cells, NANOG may also be induced by tumor microenvironments such as inflammatory cytokines and hypoxia [27, 30, 35]. NANOG-expressing cancer cells manifest certain CSC properties [6, 9, 29] and inducible NANOG expression, in a time-dependent manner, reprograms bulk cancer cells into a CSC phenotype [9]. Nevertheless, precisely how NANOG expression confers CSC traits or reprograms non-CSCs to the CSC state remains molecularly ill defined. In this study, by employing the same inducible NANOG transgene expression system in LNCaP cells that express little endogenous *NANOG* mRNA [9] and through integrative ChIP-Seq and RNA-Seq analysis combined with biological assays, we discover detailed mechanisms underlying NANOG-mediated somatic cancer cell reprogramming to the therapy-resistant and CSC state, most of which impinge on AR/FOXA1 signaling (Figure 7e).

ChIP-Seq analysis reveals overall very similar chromatin occupancy between NANOG1 and NP8, consistent with the reported similarities in the biochemical properties [14] and fibroblast-reprogramming activities [36] of the two proteins. Interestingly, ~8% of the NANOG-bound gene promoters in ESCs are also occupied by NANOG1 and/or NP8 in LNCaP cells (Figure 2c). Nevertheless, NANOG promoter binding in LNCaP cells is largely distinct from that in ESCs, forecasting potentially fundamental differences in NANOG functions in somatic cancer cells vs pluripotent cells. Indeed, NANOG genomic occupancy in LNCaP cells converges on the AR/FOXA1 genomic activity and NANOG-mediated PCa cell reprogramming dynamically represses and engages the AR transcriptional activity.

AR is the master TF that regulates the terminal differentiation of prostatic epithelial cells towards the luminal lineage. This pro-differentiation activity of AR is frequently deregulated in PCa leading to very different AR cistromes in PCa cells, especially in CRPC cells [20, 37] turning AR into an oncogenic molecule. AR generally functions in concert with several other proteins including FOXA1, NKX3.1 and NFI in PCa cells. FOXA1 functions as a ‘pioneer’ factor to promote AR activity and PCa development/progression [20, 38, 39], although it also possesses AR-independent and PCa-suppressive functions [21, 40, 41]. Strikingly, a majority of NANOG peaks in LNCaP cells co-localizes with the genomic loci occupied by AR, FOXA1, NKX3.1 and NFI members with ~70% NANOG-occupied regions overlapping with AR or FOXA1 sites and >50% of NANOG peaks overlapping with AR/FOXA1 co-occupied sites (Figure 3e). Biological assays including multispectral confocal microscopy, PLA, co-IP, GST pull-down and EMSA demonstrate that NANOG directly interacts with both AR and FOXA1. As the FOXA consensus sequence is the top binding motif for NANOG in PCa cells, and because rhNANOG protein has the potential to more strongly bind the FOXA1 genomic motif and can further interact directly with both AR and FOXA1, we speculate that NANOG may be recruited to AR and/or FOXA1 sites by preferentially binding to some forkhead response elements (FKHREs) and, either alternatively or perhaps simultaneously, by interacting with AR and FOXA1 proteins (Figure 7e). Conceptually, NANOG may bind to other variations on the FOXA1 motif with lower affinity, and differences in *cis*-element binding (taken together with variations on androgen-responsive elements (AREs)) may partly decode NANOG's localized interactome and resultant transcriptional responses on a gene-by-gene basis.

How would the NANOG genomic binding to the AR/FOXA1 loci be translated to reprogramming LNCaP cells to the castration-resistant and stem-like state that renders these cells highly resistant to androgen deprivation (for example, charcoal-dextran stripped serum, bicalutamide [9] and enzalutamide; Figure 1f, Supplementary Figure S8B) and chemotherapeutics [9]? RNA-Seq analysis, coupled with ChIP-Seq results, reveals distinct and time-dependent changes in gene expression patterns in AD and AI cells that shed light on this question (Figure 7e). Early during reprogramming (that is, d5), NANOG expression in AD LNCaP cells leads to suppression of 258 genes (in clusters 1 and 3), most of which are AR-regulated pro-differentiation molecules. This is likely achieved through a ‘competitive’ mechanism whereby NANOG occupies the FKHRE and binds directly to AR and/or FOXA1 proteins preventing AR/FOXA1-mediated transcription of these genes (Figure 7e,a). To a certain degree, this repression of pro-differentiation genes by NANOG in PCa cells is analogous to NANOG1 repression of neuroectoderm and neural crest commitment in ESCs [42]. In AD d5 cells, NANOG also activates 209 genes in clusters 2 and 6 as well as 139 genes in cluster 4. Many of these (stemness) genes may become activated by NANOG as a result of cooperation of NANOG and AR and/or FOXA1 (Figure 7e,b), as supported by genomic occupancy (Figure 5e). Such complexities in the AR cistrome and transcriptome may underlie the negative—but not mutually exclusive—relationship that we previously reported between NANOG and AR [9]. Of note, changes in the balance of FOXA1 and AR define the AR cistrome [41] (and resultant transcriptional program) and it is unclear to what extent NANOG is altering the equilibrium and occupancy of these two factors, particularly AR at half vs full AREs. Also, the presence of other TFs such as NKX3.1 and NFIX family proteins, the positions and sequences of *cis*-elements (for example, FKHREs and AREs) and the chromatin milieu, perhaps even remodeled by NANOG’s presence, may also have an impact on the transcriptional response to NANOG. Future studies should aim to elucidate the site-specific multifactorial and chromatin contributions decoding NANOG convergence on AR and FOXA1 manifesting as competitive (repressive) vs cooperative (activating).

Strikingly, NANOG-impinged gene expression profiles manifest distinct time-dependent and, in particular, castration-related changes. Specifically, the clusters 1 and 3 genes are persistently repressed, cluster 4 genes persistently activated, and cluster 2 and 6 genes first upregulated then decreased, whereas the 240 genes in cluster 5 gradually induced (Figure 5b). These results suggest that NANOG has a dominant role in suppressing the 258 cluster 1 and 3 genes regardless of whether AR signaling is present or not while the activation of the 209 cluster 2 and 6 genes likely requires AR signaling. In contrast, NANOG persistently upregulates cluster 4 genes, probably both dependent on and independently of androgen/AR signaling (Figure 7e,c). In support, there is a notable increase in ‘NP8-only’ genomic occupancy associated with the cluster 4 genes, many of which are involved in cell motility, invasion and metastasis (Figure 7e,c). Intriguingly, NANOG might be activating this cohort of genes by binding to the FKHRE-like and the NFI motifs with the assistance from NFI proteins (Figure 7e,c). Regardless, these observations provide a molecular explanation for rapid promotion of tumor cell epithelial mesenchymal transition (EMT), migration and invasion by NANOG [1, 9]. With time, continued NANOG expression in AD cells (that is, d12) upregulates the 240 cluster 5 genes, which are induced much faster and more prominently under AI conditions. These results suggest that NANOG may poise PCa cells for castration resistance, as supported by the finding that NANOG occupancy under AD conditions more closely resembles AR occupancy in AI conditions (Figure 3b). Remarkably, ~50% of the induced cluster 5 genes are related to cell cycle progression and cell division, and NANOG-expressing PCa cells proliferate much faster in enzalutamide-containing medium than control cells. Of interest, although some of these genes (for example, *CDK1* and *UBE2C*) may be directly induced by NANOG (Figure 7e,d) as supported by ChIP–qPCR analysis, most may be activated indirectly by NANOG (Figure 7e,d) through another ‘master’ TF(s) (Figure 5e), which would explain why induction of these genes is temporally late. One likely mediator could be MYC (Figure 7e,d), which is induced by NANOG *in vitro* (Supplementary Figure S7H) and *in vivo* [9]. Significant overlap between the MYC transcriptional program and the NANOG transcriptome in LNCaP cells also supports this possibility.

Integrative analysis of our genome-wide data and biological interrogations ([9] and this study) allows us to paint a complete picture of how NANOG, by temporally regulating distinct classes of genes, gradually reprograms PCa cells to the castration-resistant state and confers on them a spectrum of *de novo* phenotypes, including loss of differentiation (via repressing pro-differentiation genes), acquisition of ‘stemness’ (MYC transcriptional program and expression of stem cell genes), increased motility and invasiveness and enhanced cell proliferation, all of which are cardinal features of patient CRPC. In support, we have shown that undifferentiated (PSA^−/lo^) PCa cells, some of which possess hardcore stem cell properties being able to undergo asymmetric cell division, become significantly enriched in untreated high-grade tumors and become the predominant cell population in patient CRPC [11, 15]. Castration of some xenograft AD tumors also leads to a marked increase in PSA^−/lo^ PCa cells and in NANOG expression (Figure 1a). Interestingly, although NANOG-expressing cells increase in both LAPC9 and LAPC4 AI tumors, the LAPC4 AI tumors are characterized by increased AR and FOXA1 expression, whereas LAPC9 tumors by decreased expression of both proteins (Figure 1a). These results may suggest that in PCa cell clones progressing like LAPC4 AI, NANOG is induced to propel CRPC emergence by converging on AR/FOXA1 signaling (whether wild-type or mutant FOXA1, the latter of which has been previously reported in LAPC4 cells [43]). By contrast, in PCa cell clones resembling LAPC9 AI, NANOG might predominantly function independently of AR/FOXA1 signaling. Ongoing work is testing this interesting hypothesis. Regardless, NANOG induced in both LAPC4 and LAPC9 is required to sustain AI tumor growth.

The clinical relevance of our present findings is self-evident from comprehensive GSEA of various gene clusters. Significantly, the NANOG-repressed cluster 1 and 3 genes are associated with favorable patient survival, whereas NANOG-induced cluster 5 gene with poor patient survival. The concept that NANOG may reprogram somatic cancer cells via converging on lineage master TFs such as AR/FOXA1 is likely applicable to the NANOG functions in other tumor systems such as breast cancer. As NANOG can be induced by HIF and cytokines including interleukin-6 [1, 35], tumor microenvironments such as hypoxia and inflammation may (epigenetically) reprogram non-CSCs to the CSC state through NANOG by similarly antagonizing/engaging lineage-specific TF signaling complexes. We have further identified multiple E-box elements upstream of NP8 (−133, −486, −1 301, −1 343, −1 437 and −1 607 bp relative to the TSS [1]), suggesting that MYC and NANOG may also form a feed forward cross-regulatory loop. Finally, potential interactions in tumors among AR, NANOG and other pluripotency regulators such as SOX2 (for example, SOX2 has been implicated as an AR-repressed gene that contributes to CRPC [44] and AR has been reported to induce NANOG [45]) may well alter the landscape of the reprogramming dynamics. Future work will aim to elucidate these complicated NANOG interactions and the epigenetic mechanisms whereby NANOG maintains the CSC state.

## Materials and Methods

### Chromatin immunoprecipitation and sequencing

ChIP assay was performed by following the manufacturer’s instructions (Upstate, Charlottesville, VA, USA). In brief, LNCaP cells, including pLVX control and cells overexpressing NANOG1 or NP8 [9], were treated with DOX (500 ng ml^−1^) for 5 days before harvest. After formaldehyde fixation, lysed cell DNA was sheared by sonication and immunoprecipitated with an anti-NANOG antibody (H-155, cat# sc-33759; Santa Cruz Biotechnology, Santa Cruz, CA, USA) and protein-A beads overnight. DNA was eluted from the beads and prepared for sequencing. Sequenced DNA reads were mapped to human genome hg18 and only the reads that were mapped to unique position were retained, generating 22–26 million reads per sample of which 87–90% were mapped to human genome. The peaks were obtained using model-basedanalysis of ChIP-Seq 1.3.7.1 [46] with a scanning window set to 300 bp and a *P*-value cutoff of 1e−5 unless otherwise indicated.

### RNA-sequencing

LNCaP cells overexpressing DOX-inducible NANOG1 or NP8 relative to the pLVX control were cultured in either normal media (AD) or in charcoal-dextran stripped serum (AI) growth conditions and treated with DOX for the time intervals indicated in the figures. Cells were collected and RNA extracted using RNeasy RNA-purification kit (Qiagen, Valencia, CA, USA), including on-column DNase digestion to remove contaminating genomic DNA. One hundred nanogram of RNA was used to synthesize complemantary DNA libraries using NuGen’s Ovation RNA-Seq System following the manufacturer’s guidelines. Independent, biological triplicates of all samples were analyzed except for NP8 AD d12 that was analyzed in duplicate. The libraries were sequenced using 76 nt paired-end runs performed on an Illumina HiSeq 2000 (Illumina Inc, San Diego, CA, USA). The reads were mapped to human genome (hg18) by TopHat (version 2.0.4 for NP8/pLVX AD d12 and version 2.0.7 for other samples) and 76–91% fragments were mapped to human genome. The number of fragments in each known gene from RefSeq database (downloaded from UCSC Genome Browser on 9 March 2012) was enumerated using htseq-count from HTSeq package (version 0.5.3p9; http://www-huber.embl.de/users/anders/HTSeq/). Differential expression and statistical analyses were performed using R/Bioconductor package edgeR as described in detail in Supplementary Method. Genes with *P*-value <0.05 and fold change >1.5 were called as differentially expressed.

### Accession numbers

The NCBI GEO accession numbers are GSE74799 (for ChIP-Seq) and GSE74798 (for RNA-Seq).
