# Supplementary material for: NANOG reprograms prostate cancer cells to castration resistance via dynamically repressing and engaging the AR/FOXA1 signaling axis
Source: Cell Discov. 2016 Nov 15;2:16041–. doi: 10.1038/celldisc.2016.41 (PMC5109294; doi:10.1038/celldisc.2016.41)
Supplement: Supplementary Information [file celldisc201641-s1.pdf]

Supplemental Information

NANOG Reprograms Prostate Cancer Cells to Castration Resistance via Dynamically Repressing and Engaging the AR/FOXA1 Signaling Axis

Collene R. Jeter, Bigang Liu, Yue Lu, Hsueh-Ping Chao, Dingxiao Zhang, Xin Liu, Xin Chen, Qiuhui Li, Kiera Rycaj, Tammy Calhoun-Davis, Li Yan, Qiang Hu, Jianmin Wang, Jianjun Shen, Song Liu, and Dean G. Tang

Inventory of supplemental information

Supplemental Results
Supplemental Experimental Procedures
Supplemental References
Supplemental Figure Legends
Figure S1, related to Figure 1
Figure S2, related to Figure 2
Figure S3, related to Figure 3
Figure S4, related to Figure 4
Figure S5, related to Figure 5
Figure S6, related to Figure 5
Figure S7, related to Figure 6
Figure S8, related to Figure 7
Table S1, Primary Antibodies
Table S2, ChIP-Seq: NANOG1 & NP8 Chromatin Occupancy in LNCaP Cells
Table S3, RNA-Seq: DEGs in NANOG1 & NP8 Overexpressing LNCaP Cells AD d5
Table S4, RNA-Seq: DEGs in NP8 Overexpressing LNCaP Cells AD d12
Table S5, RNA-Seq: DEGs in NP8 Overexpressing LNCaP Cells AI d7
Table S6, RNA-Seq: DEGs in NP8 Overexpressing LNCaP Cells AI d22
Table S7, ChIP-qPCR Primers

SUPPLEMENTAL RESULTS

The NP8-Repressed 258 Genes in Clusters 1 and 3 Are AR Target Genes Associated with Normal Differentiation and ADT Response and with Better Patient Survival

Comprehensive GSEA revealed a great enrichment of Cluster 1 genes in normal human prostate (NHP) differentiated (AR^+/PSA^+) luminal cells in comparison to the AR^-PSA^- NHP basal cells (Figure 5D, a), the differentiated PSA^+ LNCaP and LAPC9 PCa cells vs. corresponding PSA^- cells (Figure 5D, b and c; See SEP for the information on data sets used in GSEA), suggesting that the Cluster 1 genes are associated with AR-regulated cellular differentiation. In support, GSEA also demonstrated that the Cluster 1 genes were enriched in primary adenocarcinomas, which oftentimes show increased AR expression and/or activity (Deng and Tang, 2015), in comparison to either normal (N; benign) prostate tissues (Figure 5D, d) or metastases (Figure 5D, e), which have very frequently lost cellular differentiation. These results would suggest that the Cluster 1 genes are associated with sensitivity to androgen deprivation therapy (ADT). Indeed, GSEA indicated that the Cluster 1 genes were enriched in cultured AD LNCaP cells (Figure 5D, f), AD xenograft (LNCaP and LAPC9) tumors (Figure 5D, g and h), and, importantly, patient primary tumors before ADT (Figure 5D, i). Strikingly, GSEA of Cluster 3 (Supplementary Figure S6A) or Cluster 1 and 3 (Supplementary Figure S6B) genes revealed an enrichment of these 258 genes in similar data sets. For example, Cluster 1 and 3 genes were enriched differentiated NHP luminal cells, PSA^+ LAPC9 cells, primary adenocarcinomas, AD LNCaP cells, AD xenograft tumors, and patient primary tumors before ADT (Supplementary Figure S6A and B). Consistent with these results, a 33-gene signature derived from the Clusters 1&3 genes was able to stratify PCa patients into the high/low risk of death groups, i.e., patients with higher expression of this gene signature had a low risk of dying (i.e., better survival) whereas patients with lower expression of this signature had a high risk of dying (i.e., poor survival) (Supplementary Figure S6G). Importantly,

this gene signature could also predict for better patient survival in an independent cohort (Supplementary Figure S6H).

Integrative Analysis of NP8 Genomic Occupancy (ChIP-Seq) and NP8-Induced DEG Clusters Reveal Distinct Mechanisms of NP8 Action

To provide potential mechanisms underlying the NP8-induced changes in gene expression, we performed integrative analysis of NP8 genomic occupancy (ChIP-Seq at AD d5) with clusters of NANOG DEGs by analyzing the genomic binding of NP8 alone or NP8 with AR and/or FOXA1 in each gene within either -50/+50 Kb (Figure 5E) or -10/+10 Kb (Supplementary Figure S6D) window. The results revealed distinct patterns of genomic occupancy that could account for NP8-induced DEG clusters. For example, for the 95 Cluster 1 genes, there was a great enrichment in genes co-occupied by NP8/AR/FOXA1 (Figure 5E; Supplementary Figure S6D) and >60% of the peaks were co-occupied by NP8 with AR and/or FOXA1 (Figure 5E). Similarly, there was a great enrichment in the Cluster 3 163 genes co-occupied by NP8/AR/FOXA1 (Figure 5E; Supplementary Figure S6D) and ~50% of the peaks were co-occupied by NP8 with AR and/or FOXA1 (Figure 5E). These results strongly suggest that NP8 occupies the genomic loci normally bound by AR and/or FOXA1 to suppress the AR/FOXA1-mediated transcription of the Cluster 1 and 3 genes. The Clusters 2 and 6 genes showed less enrichment in NP8/AR/FOXA1 co-occupancy (Figure 5E; Supplementary Figure S6D) but still ~40% and 50%, respectively, of the peaks associated with the two Clusters of genes were co-occupied by NP8 with AR and/or FOXA1 (Figure 5E). As the ChIP-Seq was performed in AD d5 LNCaP cells expressing NANOG1 or NP8 (Figure 2A) and in both NANOG1 and NP8 AD d5 cells the Clusters 2 and 6 genes were activated (Figure 5B; Supplementary Figure S5), these results suggest that a significant number of genes in these two clusters were activated by NP8 together with AR and/or FOXA1. Interestingly, the 139 genes in Cluster 4, which were persistently upregulated (Figure 5B),

were relatively enriched for genomic binding by NP8 only (Figure 5E; Supplementary Figure S6D), although coordinate NP8 and AR and/or FOXA1 targets were also apparent (Figure 5E). In all the above integrative analyses, the trends were similar using either distal (i.e., +50/-50 Kb; Figure 5E) or proximal (+10/- 10 Kb; Supplementary Figure S6D) window, although the significance was greater in the 50-Kb window, suggesting a preponderance of NANOG-mediated gene regulation at enhancers/silencers. Strikingly, the Cluster 5 DEGs were not found to be markedly enriched in any of the transcription factor categories (Figure 5E; Supplementary Figure S6D), suggesting that these genes are primarily, but not exclusively, indirect NANOG targets.

SUPPLEMENTAL EXPERIMENTAL PROCEDURES

Cell lines, Xenografts, Animals and Reagents

LNCaP prostate cancer (PCa) cells were obtained from the American Tissue Type Collection (ATCC). Xenograft human prostate tumors LAPC-4 and LAPC-9 were initially provided by Dr. C. Sawyers (Klein et al., 1997; Reiter and Sawyers, 2001) and maintained in NOD/SCID (non-obese diabetic/severe combined immunodeficiency) mice. LNCaP AD (androgen-dependent) xenograft tumors were established in our lab using early-passage cells and maintained in male NOD/SCID Interleukin-2 Receptor knockout (NSG) mice. These cell and xenograft lines were regularly authenticated by our institutional CCSG Cell Line Characterization Core using short tandem repeat (STR) analysis and checked to be free of mycoplasma contamination using the Agilent (Santa Clara, CA) MycoSensor QPCR Assay Kit (cat.#302107). NSG and NOD/SCID mice were obtained from the Jackson Laboratories (Bar Harbor, ME, USA) and maintained in standard conditions in the American Association for Accreditation of Laboratory Animal Care (AAALAC) approved MDACC Animal Facility. All experimental procedures were performed in accordance with Institutional Animal Care and Use Committee (IACUC) guidelines and approved protocols. LNCaP AI (androgen-independent) xenograft tumors were established by passaging LNCaP tumors in surgically castrated male NSG mice whereas LAPC9 and LAPC9 AI tumors in castrated NOD/SCID mice (Qin et al., 2012; Chen et al., 2016).

Tumorigenicity was measured by tumor weight and tumor incidence. In some experiments, tumor size was monitored *in vivo* using calipers to measure the tumor diameter in two dimensions and tumor volume calculated using the modified ellipsoid formula: ½ (length x width)^2. Tumors harvested were fixed in formalin and paraffin sections were cut for HE staining or IHC analysis. Basic experimental procedures for xenograft harvest, enzymatic dissociation and subcutaneous transplantation have been detailed elsewhere (Jeter et al., 2009; Jeter et al., 2011; Patrawala et al., 2006; Patrawala et al., 2007). In brief, xenograft tumors were aseptically dissected out from animals and minced into ~1 mm^3 pieces in IMDM (for LAPC-4 and LAPC-9) supplemented with 20% fetal bovine serum (FBS), or, in RPMI (for LNCaP) supplemented with 7% FBS. For AI experiments, tumors were harvested in phenol-free media with charcoal-dextran stripped serum. Tumor cells were liberated by enzymatic digestion with Accumax and dead cells and debris separated by Histopaque-1077 density gradient centrifugation. Dissociated PCa cells were used in various experiments or subject to lentiviral infection at a multiplicity of infection (MOI) 20 by overnight incubation at 37°C. Washed and resuspended cells were subcutaneously injected in 50% Matrigel in the mouse right and left flanks. All chemicals were obtained from Sigma unless specified otherwise. Primary antibodies used in this study are summarized in Supplementary Table S1.

Lentiviral shRNA and Doxycycline-Inducible Expression Systems

The pLL3.7 (control) and LL-Nanog-shRNA lentiviral vectors have been previously described (Jeter et al., 2009; Rubinson et al., 2003; Zaehres et al., 2005). The Nanog TRC-shRNA (Open Biosystems, Huntsville, AL; oligoID: TRCN000004887) has been previously described (Jeter et al., 2009). LL3.7, LL-Nanog and TRC lentiviral packaging in 293FT packaging cells was performed using 3rd generation packaging plasmids (REV, VSVg and RRE) together with the individual lentivectors. The TRIPZ-non-silencing negative control vector (cat# RHS4743), the TRIPZ-Nanog68 construct (oligo ID: V2LHS_192868) and TRIPZ-Nanog22 construct (oligo ID: V2LHS_193422) were obtained from GE Dharmacon (Lafayette, CO). TRIPZ constructs were packaged into lentivirus in 293T cells using the Trans-Lentiviral Packaging system (cat# TLP5913; GE Dharmacon). The pLVX-TetON-NANOG constructs harboring the *NANOG1* cDNA from N-TERA cells or the *NP8* coding region derived from

HPCa5 primary PCa cells (Jeter *et al.,* 2009) in pCR2.1 (Invitrogen) was subcloned into the pLVX-TetON expression vector (Clontech, Mountain View, CA, USA), as previously described (Jeter et al., 2011). NANOG overexpression was achieved using the Lenti-X Tet-ON Advanced Inducible Expression System (Clontech) per the manufacturer's instructions. LNCaP cells transduced with the pLVX-based lentivirus (pLVX empty vector control, NANOG1 or NP8) were clonally derived, as previously described (Jeter et al., 2011). pLVX constructs were packaged into lentivirus using the Lenti-X packaging system (Clontech).

Western Blotting (WB)

Basic procedures for Western blotting have been previously described (Jeter et al., 2009; Jeter et al., 2011). Briefly, whole cell lysates or nuclear extracts were prepared in RIPA buffer and run on 12% regular or 4-15% gradient SDS-PAGE gels. Proteins were transferred to nitrocellulose membrane and probed with the antibodies indicated in Supplementary Table S1.

Immunohistochemistry (IHC) of NANOG

The CRPC tissue microarray (TMA) containing 20 cases and paraffin-embedded slides from about 10 other CRPC cases (Liu et al., 2015) was kindly provided by Dr. Jiaoti Huang (Duke). For IHC, formalin fixed, paraffin-embedded tissue sections were deparaffinized and hydrated. Endogenous peroxidase activity was blocked (3% H_2O_2) and antigen retrieval was performed (10 mM citrate buffer; pH 6.0). After blocking with Biocare Blocking Reagent (Biocare), 1° antibodies (Supplementary Table S1) were incubated at appropriate dilutions for 30 min to 2 h at room temperature. Slides were washed in PBS twice and then incubated in biotinylated goat-anti-rabbit or mouse IgG (Vector Laboratories) at a 1:500 dilution for 30 min at room temperature, followed by streptavidin-conjugated horseradish peroxidase (BioGenex Laboratories Inc., San Ramon, CA) and DAB (BioGenex Laboratories Inc.) development.

Confocal Immunofluorescence (IF) and Proximity Ligation Assay (PLA)

Immunofluorescence detection of NANOG was performed via permeabilization and denaturation pretreatment (0.5% Triton X100, 0.25% sodium dodecyl sulfate) (Jeter et al., 2009). Coverslips were blocked with Background Sniper (Biocare Medical, Concord, CA, USA) for 15 min followed by primary antibody staining (all dilute to 1:250 in Dako antibody diluent, unless otherwise indicated). For triple marker analysis, sequential staining was performed with the anti-FOXA1 antibody (Abcam, goat polyclonal, cat ab5089) for 2 h at RT followed by Invitrogen chicken anti-goat AF647 for 30 min. After washing, the anti-NANOG primary antibody (Cell Signaling, rabbit monoclonal, cat# 4903S or 5232) and the anti-AR antibody (Santa Cruz, mouse monoclonal, cat# sc-7305) were applied together for 2 h at RT, followed by simultaneous staining with goat-anti-rabbit AF488 and goat-anti-mouse AF564, both from Invitrogen. Following DAPI staining (300 nM) for 10 min, coverslips were cleared in ddH2O prior to mounting in PermaGold Antifade Mounting Agent (Invitrogen, Carlsbad, CA). Duolink Proximity Ligation Assay (Sigma) was performed using simultaneous staining with the anti-NANOG primary antibody (Cell Signaling, rabbit monoclonal cat# 5232), and the anti-FOXA1 antibody (Abcam, goat polyclonal, cat# ab5089) or anti-AR antibody (Santa Cruz, mouse monoclonal, cat# sc-7305) according to the manufacturer's recommendations. IF images were acquired on a Zeiss LSM510 META confocal microscope using the apo/plan 63X or 100X objective as indicated. For the proximity ligation assay, z-stack images (1 μm optical sections) were integrated into a single composite 2D image.

Response of NANOG-Overexpressing LNCaP Cells to MDV3100 (Enzalutamide)

To assess the effects of NANOG on enzalutamide-induced growth arrest of PCa cells, LNCaP-pLVX, -NANOG1 or -NP8 cells were plated at 50K/well in a 24-well plate + 1 μg/mL Dox for 48 h to induce NANOG expression. The medium was subsequently removed (i.e., at day 0) and replaced with phenol-free RPMI + 5% CDSS and 40 μM MDV3100 for the indicated time. Cells were trypsinized and trypan blue-excluding viable cells counted using a hemacytometer.

EdU Proliferation Assay and Flow Cytometry

LNCaP cells overexpressing NANOG1 or NP8 (vs. pLVX) were cultured in 20 μM MDV3100 (and phenol-free RPMI and CDSS) for 30 d. Cells were replated at 100K/well in a 6-well dish prior to siRNA transfection. The following day, RNAiMAX (Thermofisher) was used to transfect cells with 100 nM experimental siRNA (anti-MYC, Origene cat# SR03025A/B/C and anti-UBE2C, Dharmacon OnTarget Pool cat# J004693-00-0005) vs. siCTRL (NC1, Origene cat # SR30004). Proliferation was determined 3 d later using the ClickIt Edu (Invitrogen, cat#) kit per manufacturer's instructions. Flow analysis was performed using a FACSAria flow cytometer (BD Biosciences, San Jose, CA, USA).

ChIP-Seq and Bioinformatic Analysis

Mapping of reads: Sequenced DNA reads were mapped to human genome hg18 using ELAND from Illumina analysis pipeline and only the reads that were mapped to unique position were retained. 22-26 million reads were generated per sample. 87-90% reads were mapped to human genome, with 66-70% uniquely mapped. To avoid PCR bias, for multiple reads that were mapped to the same genomic position, only one copy was retained for further analysis. 13-16 million reads were finally used in peak calling and downstream analyses.

Peak calling: Peaks of NANOG1 and NP8 were detected by MACS (version 1.3.7.1) (Zhang et al., 2008). The window size was set as 300 bp and the *p*-value cutoff was 1e-5. NANOG1 peaks were initially called by comparing pNANOG1+Dox+NgIP (i.e., pLVX-NANOG1 cells treated with Dox and subjected to NANOG IP) to pNANOG1-Dox+NgIP (i.e., pLVX-NANOG1 cells without Dox and subjected to NANOG IP). NP8 peaks were initially called by comparing pNP8+Dox+NgIP to pNP8-Dox+NgIP. Then for both NANOG1 and NP8, the peaks that were not significant when using pLVX+Dox+IgG and pLVX+Dox+NgIP as controls were removed. 14,331 and 14,449 peaks were retained for NANOG1 and NP8, respectively.

Distribution of NANOG1/NP8 peaks: Each peak was assigned to the gene that has the closest transcription start site (TSS) to it. Then the peak was classified by its location to the gene: 5' distal (-15Kb to -5Kb from TSS), promoter (-5Kb to +0.5Kb from TSS), exon, intron, 3' proximal (-0.5Kb to +5Kb from TES), 3' distal (+5Kb to +15Kb from TES) and gene desert. For the Venn diagram of promoter occupancy by NANOG, the promoter region of a gene was defined as -8Kb to +2Kb from TSS (Boyer et al., 2005). The genes used to annotate the peaks are the RefSeq genes (Pruitt et al., 2014) downloaded from UCSC genome browser (http://genome.ucsc.edu/) on December 13, 2010.

Correlation with histone methyl marks: The following ChIP-grade antibodies were used to interrogate the epigenetic status of regions of NANOG occupancy in LNCaP cells: anti-H3K4me1 (Abcam, cat# ab8895), H3K4me3 (Millipore, cat# 04-745) and H3K27me3 (Millipore, cat# 07-449). 5 μg fixed and sonication-sheared DNA from untreated LNCaP cells was co-incubated with the indicated antibody and protein-A beads for 3 h. The DNA was eluted from the beads and prepared for sequencing. H3K4me2 data was acquired from published data (GSE20042/GSM503905). Bioinformatic processing

for histone mark ChIP-Seq signals are performed as described above, subtracting input signal as the baseline and detailed procedures for histone mark ChIP-Seq experiments will be presented elsewhere.

Public ChIP-Seq data processing: All the public ChIP-Seq data were downloaded from GEO (Gene Expression Omnibus) website (http://www.ncbi.nlm.nih.gov/geo/) and included: NANOG in ESCs (GEO: GSE21200/GSM518374), FOXA1 in LNCaP cells (GEO: GSE28264; AD: GSM699635 and AI: GSM699634), AR in LNCaP cells (GEO: GSE28264; AD: GSM699631 and AI: GSM699630), NKX3.1 in LNCaP cells (GEO: GSE28264; AD: GSM699633 and AI: GSM699632), and CTCF control in LNCaP cells (GEO: GSE38684/GSM947528). For FOXA1, AR, NKX3.1 and H3K4me2, the mapped files were downloaded directly. For NANOG in ESCs and CTCF in LNCaP, raw sequences were downloaded and mapped to hg18 using bowtie (version 0.12.8) (Langmead et al., 2009). The peaks were called by MACS (version 1.3.7.1) (window size 300 bp, *p*-value cutoff 1e-5) using the corresponding input as control.

Landscape of ChIP-Seq signal: Each read was extended by 150 bp to its 3' end. The number of reads on each genomic position was rescaled to normalize the total number of mapped reads to 10M and averaged over every 10 bp window. The normalized values were displayed in UCSC genome browser.

Heat map and distribution analysis of ChIP-Seq signals proximal to NP8 peaks: Signal distribution heat map analysis of ChIP-Seq peaks (NP8, AR, FOXA1, and NKX3.1) were centered on NP8, +/- 10 kb from the peak using R function heatmap.2. The signal distribution 10 kb upstream and downstream from each NP8 peak summit was subdivided into 250 bp bins. For each ChIP-Seq sample, the RPKM (reads per kilobase transcript per million reads) value for each bin was calculated. The RPKM values were then averaged over all peaks to generate average profile or plotted in heat map by R function heatmap.2. Analysis of histone-methyl marks in chromatin regions corresponding to NANOG peaks was performed using the 10 Kb upstream to 10 Kb downstream from each NANOG peak submit (promoter regions or non-promoter associated) subdivided into 250 bp bins. In each bin, the number of tags was normalized to RPKM and the RPKM value was averaged over all the peaks and then plotted.

Colocalization heatmap: The peaks of all factors shown in the heatmap were merged into a superset of binding sites. The RPKM values of each factor in these merged sites were calculated. Then the Pearson's Correlation Coefficient between every pair of factors using these RPKM values was calculated and plotted in heatmap by R function heatmap.2. Hierarchical clustering was performed by hclust function in R using Euclidean distance and average linkage clustering method. The color of each cell indicates the Pearson correlation of co-localization behavior of each pair of samples calculated from the observed versus expected overlap matrix of all pairs.

Motif analysis: For *de novo* motif analysis, peaks were called via MACS using a more stringent *p*-value cutoff of 1e-10. Motif discovery was performed using MEME (Multiple EM for Motif Elicitation; (Bailey et al., 2006)) version 4.7.0 and motifs discovered by MEME using option -maxlen 25. The 100-bp sequences flanking the summit of the top 800 peaks (by *p*-value) in NANOG1 or NP8 were used to search for occurring motifs. Identification of centrally enriched motifs by CentriMo and match of identified motifs to known motifs by Tomtom were performed using MEME-ChIP (Machanick and Bailey, 2011) from MEME Suite (version 4.9.0) (Bailey et al., 2009). The sequences of +/- 500 bp from the summit of peaks were taken as input and MEME was set to run on 600 randomly picked peaks. MAST from MEME suite was used to identify the existence of motifs in peaks (the *p*-value cutoff was set at 1e-4). AR motifs included the full AR motif Jaspar ID MA0007.1 and half motifs residues 1-11 [ARE A] and 12-22 [ARE B].

RNA-Seq, Data Processing and Bioinformatics

Experiment: Basic procedures for RNA-Seq and data processing have recently been reported (Zhang et al., 2016) and described in main Text. The libraries were sequenced using 2x76 base paired end protocol on Illumina HiSeq 2000 instrument. Three biological replicates were prepared for each

condition, except NP8/pLVX AD d12, which had two biological replicates. 26-40 million pairs of reads were generated per sample. Each pair of reads represents a cDNA fragment from the library. The reads were mapped to human genome (hg18) by TopHat (version 2.0.4 for NP8/pLVX AD d12 and version 2.0.7 for other samples) (Kim et al., 2013). 76-91% fragments were mapped to human genome. ***Differential expression:*** The number of fragments in each known gene from RefSeq database (Pruitt et al., 2014) (downloaded from UCSC Genome Browser on March 9, 2012) was enumerated using htseq-count from HTSeq package (version 0.5.3p9) http://www-huber.embl.de/users/anders/HTSeq/. Differential expression analysis was performed separately for different AI/AD and short/long-term conditions. Genes with less than 10 fragments in all the samples were first removed and then differential expression of NANOG1 or NP8 vs. pLVX (control) was statistically assessed by R/Bioconductor package edgeR (Robinson et al., 2010) (version 3.0.4 for AD d12 and version 3.0.8 for other conditions). The samples from AD d12 showed apparent batch effect, thus the edgeR code was modified to remove the batch effect following edgeR users guide. For other conditions, edgeR classic approach was used. Genes with $p< 0.05$ and fold change >1.5 were called as differentially expressed. ***Gene clustering and heatmap:*** Hierarchical clustering was performed on differentially expressed genes from any of the five comparisons (NANOG1/NP8 vs. pLVX under AD/AI and short/long term) using the log2 ratio values by hclust function in R. The log2 ratio values in each row were rescaled so that the sum of the squares of the values is 1.0. Euclidean distance and ward clustering method were used to construct the dendrogram. The heatmap was plotted by heatmap.2 function in R. According to the dendrogram, the genes were classified into 11 groups (Supplementary Figure S5).

IPA, DAVID and GSEA

For Gene Ontology (GO) analysis, IPA (Qiagen, Valencia, CA) and DAVID version 6.7 were used with gene symbols. GSEA was carried out by using the curated gene sets (C2) of the Molecular Signature Database (MSigDB) version 4.0 provided by the Broad Institute (http://www.broad.mit.edu/gsea/) (Subramanian et al., 2005). In general, we followed the standard procedure as described by GSEA user guide (http://www.broadinstitute.org/gsea/doc/GSEAUserGuideFrame.html). The FDR for GSEA is the estimated probability that a gene set with a given NES (normalized enrichment score) represents a false positive finding, and an FDR<0.25 is considered to be statistically significant for GSEA.

Most gene signatures used in this study were obtained from MSigDB and described earlier (Zhang et al., 2016). RNA-Seq data sets and/or gene signatures employed to perform GSEA of our NANOG-induced LNCaP transcriptomic profile and DEGs included: **1)** an RNA-Seq data set in normal human prostate luminal and basal cells (Zhang et al., 2016); **2)** RNA-Seq data sets in purified PSA^- and PSA^+ LNCaP and LAPC9 cells (Liu X, et al., manuscript in preparation); **3)** an RNA profiling data set comparing primary prostate tumors (T) vs. benign/normal (N) tissues or metastatic samples (M) (Varambally et al., 2005); **4)** an RNA-Seq data set comparing the regular LNCaP cells (AD) vs. castration resistant (AI) LNCaP cells (Wang et al., 2014); **5)** RNA-Seq data sets in AD vs. AI LNCaP and LAPC9 xenograft tumors (Chen X et al., manuscript in preparation); and **6)** an RNA-Seq data comparing the transcriptomes of patient prostate tumors before vs. after ADT (Rajan et al., 2014).

Oncomine Concept Analysis

The gene lists from hierarchical clustering (Supplementary Figure S5; Figure 5B) served as the custom concepts in *Oncomine* Concept Analysis for down-regulated and unregulated profiles for Cluster 1&3 and Cluster 5 genes, respectively. The comparison between tumors (T) to normal (N) utilized 16 data

sets (listed as 1-16 in Supplementary Figure S6E and F), which were Arredouani et al., 2009, Grasso et al., 2012, Holzbeierlein et al., 2004, Lapointe et al., 2004, LaTulippe et al., 2002, Liu et al., 2006, Luo et al., 2002, Magee et al., 2001, Singh et al., 2002, Taylor et al., 2010, Tomlins et al., 2007, Vanaja et al., 2003, Varambally et al., 2005, Wallace et al., 2008, Welsh et al., 2001, and Yu et al., 2004. The comparison between metastasis and primary tumors employed 13 data sets in Oncomine, which were Chandran et al., 2007, Grasso et al., 2012, Holzbeierlein et al., 2004, Lapointe et al., 2004, LaTulippe et al., 2002, Magee et al., 2001, Ramaswamy et al., 2001, Ramaswamy et al., 2003, Tamura et al., 2007, Taylor et al., 2010, Vanaja et al., 2003, Varambally et al., 2005, and Yu et al., 2004).

Derivation of Gene Signatures and Patient Survival Analyses

RNA-Seq analysis revealed persistent repression of genes in Clusters 1 and 3 and time-dependent induction of genes in Cluster 5. We aimed to derive a gene signature from Clusters 1/3 and a gene signature from Cluster 5 genes and then to determine whether these signatures can stratify PCa patients into high- vs. low-risk of death groups and further can predict for patient survival. A gene signature consists of a set of genes that are predictive to a disease outcome, and an algorithm that transforms their measured expression into a clinically relevant statement, for example, combining the gene expression values to a single predictor to classify the patient to one of the high/low risk groups (Buyse et al., 2006). To develop gene signatures based on the DEGs in Clusters 1/3 and Cluster 5, respectively, we downloaded, from the *Oncomine* database (Liu et al., 2015), three independent cohorts (i.e., Setlur with 363 samples, Taylor with 140 samples, and Glinsky with 79 samples) that have both gene expression and patient survival information. In both Setlur and Taylor cohorts, we used overall survival status (Alive/Dead) and time as our outcomes. In Glinsky cohort, the Recurrence (yes/no) and time were used instead, as there is no overall survival information available. The genes that are shared between our gene sets (i.e., Clusters 1/3 and Cluster 5) and those measured in all three cohorts are retained, which resulted in 33 genes for Clusters 1/3, and 58 genes for Cluster 5. We used the largest cohort (Setlur) as our training set to derive the gene signature and the rest two cohorts (Taylor and Glinsky) as independent testing sets. Specifically, the algorithm for creating a predictor involves a numeric score and a threshold to classify the subject to a binary (high/low) risk class variable based on the score. The numeric score was calculated based on a linear combination of expression values of the genes in the signature. The risk coefficients, i.e., the weights of each gene, are estimated using the 'pairwise' non-parametric linear combination method (Yan et al., 2015). The risk coefficients was optimized to maximize the overall discrimination power (measured by Area under Curve [AUC] of the combined predictor) to separate the dead and alive subgroups in the training set. The threshold of the classification is determined by maximizing the accuracy of the combined predictor in the training set. The accuracy of the derived predictor is evaluated in the independent test sets.

Integrative Analysis of ChIP-Seq and RNA-Seq Data on Gene Clusters

Integrative analysis of ChIP-Seq and RNA-Seq was used to determine coordinate genomic occupancy and transcriptional regulation (Figure 5E and Supplementary Figure S6D). Categorization of ChIP-Seq occupancy into sites occupied by all 3 factors (NP8, AR and FOXA1), pairs of factors (NP8 and AR or FOXA1) or NP8 only was performed by single nucleotide (or more) overlap in peak signals (as in Figure 3C). RNA-Seq DEGs (up and down 1.5X and $P<0.05$) were extracted from the unsupervised cluster heatmap (Figure 5B). Comparative analyses were performed using all NP8 peaks in the given category using the closest gene in a 50 Kb window (Figure 5E) or all the genes in a 10 Kb window (Supplementary Figure S6D) and a total number of 21,706 possible genes, excluding the genes shorter

than 200 bp not reliably detectable by RNA-Seq. The significance of the observed vs. expected overlap was calculated by Fisher's Exact test.

Co-Immunoprecipitation (Co-IP) Experiments

The cytoplasmic fraction and nuclear extracts (NE) were prepared with Nuclear and Cytoplasmic Extraction kit per manufacturer's instruction (NE-PER™ Nuclear and Cytoplasmic Extraction Reagents, Thermo Scientific, Cat. 78833). 800 μg NE was incubated with anti-AR (Abcam, cat#: ab74272) or anti-FOXA1 (Abcam, cat#: ab23738) antibodies overnight at 4°C. Rabbit polyclonal IgG (Abcam, cat#: ab27478) was used as the antibody control. The solution was then incubated with protein A-agarose beads (Sigma, MO, USA) for 1 h at 4°C, after which the beads were washed three times with RIPA buffer. Proteins bound to protein A-agarose were eluted with SDS-PAGE loading buffer and boiled for 8 min and subjected to SDS-PAGE. Gels were transferred onto an immobilon-P transfer membrane (Millipore, Bedford, MA), which was then blocked with 5% non-fat dried milk in TBST (10 mM Tris-HCl, 150 mM NaCl and 0.1% Tween-20) for 1 h at room temperature, and incubated overnight at 4°C with primary antibodies (anti-AR mouse mAb, Santa Cruz, cat# sc-7305; anti-FOXA1 goat polyclonal Ab, Abcam, cat#: ab5089; or anti-Nanog mouse mAb, eBioscience, cat#: 145768). Membranes were washed three times with TBST buffer, incubated for 1 h with secondary antibodies (1:1000), and developed with ECL Plus WB detection reagent (PerkinElmer).

Preparation of Soluble Recombinant Human NANOG (rhNANOG) Protein

Human NP8 cDNA was subcloned into pET-28a (+) expression vector to generate His-tagged fusion protein (Liu et al, 2014). rhNP8 was expressed in *E. coli* B21. To obtain a large amount of soluble rhNP8, bacterial pellets were sonicated 8 times with 30 seconds pulses. The sonication solution was centrifuged at 16,000 rpm for 30 min at 4 ℃ and the pellets were washed with cold PBS three times. The precipitated material was the inclusion body of rhNP8 protein, which was dissolved in a buffer (1.5 M NaCl, 10 mM Tris-HCl, pH 7.0) containing 7 M urea. Subsequently, the solution was subjected to dialysis in the same buffer containing 1 M, 0.5 M, 0.1 M, and 0.05 M urea. The dialysis solution was subjected to SDS-PAGE to quantify soluble NP8 protein.

GST Fusion Protein Preparation and Purification

Full-length cDNAs of human AR and human FOXA1 were synthesized by GenScript (Piscataway Township, NJ) and then subcloned into the GST-fusion expression vector pGEX-6p-1. The fusion proteins of GST-AR and GST-FOXA1 were overexpressed in *E. coli* B21 cells via induction by isopropyl-β-d-thiogalactopyranoside (IPTG, 0.4 mM). Washed bacterial cells were resuspended in 2 ml of phosphate-buffered saline (PBS) and subsequently broken by eight 30-s sonicator pulses (30% duty) on ice with a Digital Sonifier cell disruptor (SmithKline Corp.). The resulting lysate was centrifuged for 30 min at 16,000 rpm at 4 °C. The GST fusion proteins were then batch-purified from extracts by binding to Glutathione-Sepharose 4B beads (GE Healthcare) and washed in PBS according to the manufacturer's instructions. The purified proteins were eluted from the beads with 30 mM glutathione, 50 mM Tris-HCl, pH 7.5, 120 mM NaCl, and 2% glycerol.

GST Pull-down Assays

GST (control), or GST–AR or GST–FOXA1 ($C_2H_2 \rightarrow A_2H_2$) fusion proteins on glutathione–Sepharose™ 4B beads were co-incubated with the soluble rhNP8 (above) in 1 ml of mild lysis buffer

(10 mM Tris-HCI, pH 7.5, 150 mM NaCI, 5 mM EDTA, and 1% Triton). Beads with their associated proteins were washed 3X with mild lysis buffer. Bound proteins were separated by SDS/PAGE and transferred to PVDF membrane, which was blocked with 5% non-fat dried milk in TBST (10 mM Tris-HCl, 150 mM NaCl and 0.1% Tween-20) for 1 h at room temperature, and incubated overnight at 4℃ with an anti-NANOG antibody (Cell Signaling) and subsequent WB analysis.

Electrophoretic Mobility Shift Assays (EMSA)

EMSA was performed according the instruction of LightShift Chemiluminescent EMSA Kit (Thermo SCIENTIFIC, Cat#: 20148). The biotin-labeled and unlabeled consensus probes (from UBE2C gene) were prepared by annealing either two biotin-labeled or unlabeled complementary primers containing FOXA1 binding motifs (5'-CTTAGTGTTTATTTCAAGCT-3' (sense strand), and 5'-AGCTTGAAATAAACACTAAG-3' (anti-sense strand)). These primers were synthesized by Sigma. The mixture with equal amount of the two complementary primers were heated in the binding buffer (20 mM Tris-HCl, pH 7.5, 150 mM NaCl) for 10 minutes at 100^0C, then annealed by naturally cooling down to room temperature and the resulting solution contained double-stranded probes and single-stranded primers. For EMSA, 100 ng recombinant GST-FOXA1 and His-tagged NANOG protein were used in binding reactions with the biotin-labeled consensus probe. Specificity was determined by the prior addition of 300-fold excess of unlabeled competitor consensus probe. In some experiments, different ratios of FOXA1 protein vs. NANOG protein were added to the reaction mixture to determine the competitive binding to the FOXA1 binding motif between FOXA1 and NANOG. The reactions were separated on 4-20% non-denaturing acrylamide gels in 0.5X TBE buffer. After the binding mixture was transferred to the Nylon membrane (Bio-Rad), biotin-labeled DNA was detected by chemiluminescence.

Chromatin Immunoprecipitation qPCR (ChIP-PCR)

ChIP was performed using the R&D anti-NANOG antibody (AF1997), formaldehyde cross-linked and sonicated DNA from pLVX-, NANOG1- or NP8- LNCaP cells (cultured in the presence of Dox under AD or AI [20 μM MDV3100] conditions, as described) and a Chromatin Immunoprecipitation Assay Kit (Millipore, cat# 17-295). Basic procedures for qPCR have been previously described (Jeter et al., 2011). In brief, primers designed against NANOG-occupied loci in proximity to genes of interest (see Supplementary Figure S8) and chromatin-immunoprecipitated template DNA (or input DNA, as control) were used along with iTaq Universal SYBR Green One-step Kit (Bio-Rad, cat# 172-5151) for qPCR. Reactions were run on an ABI Prism 7900HT (Applied Biosystems). The experimental Ct (cycle threshold) was calibrated against that of input control product. All amplifications were performed in duplicate. The ΔΔCt method was used to determine the amount of immunoprecipitated DNA relative to that of the pLVX control with the chromatin levels of a particular locus in NANOG1 and NP8 overexpressing cells presented as fold change relative to the pLVX control cells (1-fold, 100%).

Statistical Analysis

All statistical analyses (with the exception of bioinformatics analyses, described separately above) were performed in Excel using unpaired Student's *t*-test, ANOVA (*F*-test), or χ^2 test depending on the nature of comparisons and type of data.

SUPPLEMENTAL REFERENCES

Arredouani, M. S., B. Lu, M. Bhasin, M. Eljanne, W. Yue, J. M. Mosquera, G. J. Bubley, V. Li, M. A. Rubin, T. A. Libermann, and M. G. Sanda. (2009). Identification of the transcription factor single-minded homologue 2 as a potential biomarker and immunotherapy target in prostate cancer. *Clin Cancer Res* 15 (18):5794-802.

Bailey, T.L., Boden, M., Buske, F.A., Frith, M., Grant, C.E., Clementi, L., Ren, J., Li, W.W., and Noble, W.S. (2009). MEME SUITE: tools for motif discovery and searching. Nucleic Acids Res *37*, W202-208.

Bailey, T.L., Williams, N., Misleh, C., and Li, W.W. (2006). MEME: discovering and analyzing DNA and protein sequence motifs. Nucleic Acids Res *34*, W369-373.

Boyer, L.A., Lee, T.I., Cole, M.F., Johnstone, S.E., Levine, S.S., Zucker, J.P., Guenther, M.G., Kumar, R.M., Murray, H.L., Jenner, R.G., *et al.* (2005). Core transcriptional regulatory circuitry in human embryonic stem cells. Cell *122*, 947-956.

Buyse M, Loi S, van't Veer L, Viale G, Delorenzi M, Glas AM, d'Assignies MS, Bergh J, Lidereau R, Ellis P, *et al*., (2006). Validation and clinical utility of a 70-gene prognostic signature for women with node-negative breast cancer. J Natl Cancer Inst. *98,* 1183-1192.

Chandran, U. R., C. Ma, R. Dhir, M. Bisceglia, M. Lyons-Weiler, W. Liang, G. Michalopoulos, M. Becich, and F. A. Monzon. (2007). Gene expression profiles of prostate cancer reveal involvement of multiple molecular pathways in the metastatic process. *BMC Cancer* 7:64.

Chen X, Li Q, Liu X, Liu C, Liu R, Rycaj K, Zhang D, Liu B, Jeter C, Calhoun-Davis T, *et al*. (2016). Defining a population of stem-like human prostate cancer cells that can generate and propagate castration-resistant prostate cancer (CRPC). Clin Cancer Res. Apr 8. pii: clincanres.2956.2015. [Epub ahead of print]

Deng Q, and Tang DG. (2015). Androgen receptor and prostate cancer stem cells: biological mechanisms and clinical implications. Endocr Relat Cancer *22*, T209-220.

Grasso, C. S., Y. M. Wu, D. R. Robinson, X. Cao, S. M. Dhanasekaran, A. P. Khan, M. J. Quist, X. Jing, R. J. Lonigro, J. C. Brenner, I. A. Asangani, B. Ateeq, S. Y. Chun, J. Siddiqui, L. Sam, M. Anstett, R. Mehra, J. R. Prensner, N. Palanisamy, G. A. Ryslik, F. Vandin, B. J. Raphael, L. P. Kunju, D. R. Rhodes, K. J. Pienta, A. M. Chinnaiyan, and S. A. Tomlins. (2012). The mutational landscape of lethal castration-resistant prostate cancer. *Nature* 487 (7406):239-43.

Holzbeierlein, J., P. Lal, E. LaTulippe, A. Smith, J. Satagopan, L. Zhang, C. Ryan, S. Smith, H. Scher, P. Scardino, V. Reuter, and W. L. Gerald. (2004). Gene expression analysis of human prostate carcinoma during hormonal therapy identifies androgen-responsive genes and mechanisms of therapy resistance. *Am J Pathol* 164 (1):217-27.

Jeter, C.R., Badeaux, M., Choy, G., Chandra, D., Patrawala, L., Liu, C., Calhoun-Davis, T., Zaehres, H., Daley, G.Q., and Tang, D.G. (2009). Functional evidence that the self-renewal gene NANOG regulates human tumor development. Stem Cells *27*, 993-1005.

Jeter, C.R., Liu, B., Liu, X., Chen, X., Liu, C., Calhoun-Davis, T., Repass, J., Zaehres, H., Shen, J.J., and Tang, D.G. (2011). NANOG promotes cancer stem cell characteristics and prostate cancer resistance to androgen deprivation. Oncogene.

Kim, D., Pertea, G., Trapnell, C., Pimentel, H., Kelley, R., and Salzberg, S.L. (2013). TopHat2: accurate alignment of transcriptomes in the presence of insertions, deletions and gene fusions. Genome Biol *14*, R36.

Klein, K.A., Reiter, R.E., Redula, J., Moradi, H., Zhu, X.L., Brothman, A.R., Lamb, D.J., Marcelli, M., Belldegrun, A., Witte, O.N., *et al.* (1997). Progression of metastatic human prostate cancer to androgen independence in immunodeficient SCID mice. Nat Med *3*, 402-408.

Langmead, B., Trapnell, C., Pop, M., and Salzberg, S.L. (2009). Ultrafast and memory-efficient alignment of short DNA sequences to the human genome. Genome Biol *10*, R25.

Lapointe, J., C. Li, J. P. Higgins, M. van de Rijn, E. Bair, K. Montgomery, M. Ferrari, L. Egevad, W. Rayford, U. Bergerheim, P. Ekman, A. M. DeMarzo, R. Tibshirani, D. Botstein, P. O. Brown, J. D. Brooks, and J. R. Pollack. (2004). Gene expression profiling identifies clinically relevant subtypes of prostate cancer. *Proc Natl Acad Sci U S A* 101 (3):811-6.

LaTulippe, E., J. Satagopan, A. Smith, H. Scher, P. Scardino, V. Reuter, and W. L. Gerald. (2002). Comprehensive gene expression analysis of prostate cancer reveals distinct transcriptional programs associated with metastatic disease. *Cancer Res* 62 (15):4499-506.

Liu, B., Badeaux, M.D., Choy, G., Chandra, D., Shen, I., Jeter, C.R., Rycaj, K., Lee, C.F., Person, M.D., Liu, C., *et al*. (2014). Nanog1 in NTERA-2 and recombinant NP8 from somatic cancer cells adopt multiple protein conformations and migrate at multiple M.W species. PLoS One *9*, e90615.

Liu, P., S. Ramachandran, M. Ali Seyed, C. D. Scharer, N. Laycock, W. B. Dalton, H. Williams, S. Karanam, M. W. Datta, D. L. Jaye, and C. S. Moreno. (2006). Sex-determining region Y box 4 is a transforming oncogene in human prostate cancer cells. *Cancer Res* 66 (8):4011-9.

Liu X, Chen X, Rycaj K, Chao HP, Deng Q, Jeter C, Liu C, Honorio S, Li H, Davis T, *et al*. (2015). Systematic dissection of phenotypic, functional, and tumorigenic heterogeneity of human prostate cancer cells. Oncotarget *6*, 23959-23986.

Luo, J. H., Y. P. Yu, K. Cieply, F. Lin, P. Deflavia, R. Dhir, S. Finkelstein, G. Michalopoulos, and M. Becich. 2002. Gene expression analysis of prostate cancers. *Mol Carcinog* 33 (1):25-35.

Machanick, P., and Bailey, T.L. (2011). MEME-ChIP: motif analysis of large DNA datasets. Bioinformatics *27*, 1696-1697.

Magee, J. A., T. Araki, S. Patil, T. Ehrig, L. True, P. A. Humphrey, W. J. Catalona, M. A. Watson, and J. Milbrandt. (2001). Expression profiling reveals hepsin overexpression in prostate cancer. *Cancer Res* 61 (15):5692-6.

Patrawala, L., Calhoun, T., Schneider-Broussard, R., Li, H., Bhatia, B., Tang, S., Reilly, J.G., Chandra, D., Zhou, J., Claypool, K., *et al.* (2006). Highly purified CD44+ prostate cancer cells from xenograft human tumors are enriched in tumorigenic and metastatic progenitor cells. Oncogene *25*, 1696-1708.

Patrawala, L., Calhoun-Davis, T., Schneider-Broussard, R., and Tang, D.G. (2007). Hierarchical organization of prostate cancer cells in xenograft tumors: the CD44+alpha2beta1+ cell population is enriched in tumor-initiating cells. Cancer Res *67*, 6796-6805.

Pruitt, K.D., Brown, G.R., Hiatt, S.M., Thibaud-Nissen, F., Astashyn, A., Ermolaeva, O., Farrell, C.M., Hart, J., Landrum, M.J., McGarvey, K.M., *et al.* (2014). RefSeq: an update on mammalian reference sequences. Nucleic Acids Res *42*, D756-763.

Qin, J., Liu, X., Laffin, B., Chen, X., Choy, G., Jeter, C.R., Calhoun-Davis, T., Li, H., Palapattu, G.S., Pang, S., *et al.* (2012). The PSA$^{-/lo}$ prostate cancer cell population harbors self-renewing long-term tumor-propagating cells that resist castration. Cell Stem Cell *10*, 556-569.

Ramaswamy, S., K. N. Ross, E. S. Lander, and T. R. Golub. (2003). A molecular signature of metastasis in primary solid tumors. *Nat Genet* 33 (1):49-54.

Ramaswamy, S., P. Tamayo, R. Rifkin, S. Mukherjee, C. H. Yeang, M. Angelo, C. Ladd, M. Reich, E. Latulippe, J. P. Mesirov, T. Poggio, W. Gerald, M. Loda, E. S. Lander, and T. R. Golub. (2001). Multiclass cancer diagnosis using tumor gene expression signatures. *Proc Natl Acad Sci U S A* 98 (26):15149-54.

Rajan, P., Sudbery IM, Villasevil ME, Mui E, Fleming J, Davis M, Ahmad I, Edwards J, Sansom OJ, Sims D, *et al.* (2014). Next-generation sequencing of advanced prostate cancer treated with androgen-deprivation therapy. Eur. Urol. *66*, 32-39.

Reiter, R.E., and Sawyers, C.L. (2001). Xenograft models and the molecular biology of human prostate cancer. In Prostate cancer: Biology, Genetics, and the New Therapeutics, L.W.K. Chung, W.B. Isaacs, and J.W. Simons, eds. (Totowa, NJ: Humana Press Inc.), pp. 163-174.

Robinson, M.D., McCarthy, D.J., and Smyth, G.K. (2010). edgeR: a Bioconductor package for differential expression analysis of digital gene expression data. Bioinformatics *26*, 139-140.

Rubinson, D.A., Dillon, C.P., Kwiatkowski, A.V., Sievers, C., Yang, L., Kopinja, J., Rooney, D.L., Zhang, M., Ihrig, M.M., McManus, M.T.*, et al.* (2003). A lentivirus-based system to functionally silence genes in primary mammalian cells, stem cells and transgenic mice by RNA interference. Nat Genet *33*, 401-406.

Singh, D., P. G. Febbo, K. Ross, D. G. Jackson, J. Manola, C. Ladd, P. Tamayo, A. A. Renshaw, A. V. D'Amico, J. P. Richie, E. S. Lander, M. Loda, P. W. Kantoff, T. R. Golub, and W. R. Sellers. (2002). Gene expression correlates of clinical prostate cancer behavior. *Cancer Cell* 1 (2):203-9.

Subramanian, A. Tamayo P, Mootha VK, Mukherjee S, Ebert BL, Gillette MA, Paulovich A, Pomeroy SL, Golub TR, Lander ES, *et al.* (2005). Gene set enrichment analysis: a knowledge-based approach for interpreting genome-wide expression profiles. Proc. Natl Acad. Sci. USA *102*, 15545-15550.

Tamura, K., M. Furihata, T. Tsunoda, S. Ashida, R. Takata, W. Obara, H. Yoshioka, Y. Daigo, Y. Nasu, H. Kumon, H. Konaka, M. Namiki, K. Tozawa, K. Kohri, N. Tanji, M. Yokoyama, T. Shimazui, H. Akaza, Y. Mizutani, T. Miki, T. Fujioka, T. Shuin, Y. Nakamura, and H. Nakagawa. (2007). Molecular features of hormone-refractory prostate cancer cells by genome-wide gene expression profiles. *Cancer Res* 67 (11):5117-25.

Taylor, B. S., N. Schultz, H. Hieronymus, A. Gopalan, Y. Xiao, B. S. Carver, V. K. Arora, P. Kaushik, E. Cerami, B. Reva, Y. Antipin, N. Mitsiades, T. Landers, I. Dolgalev, J. E. Major, M. Wilson, N. D. Socci, A. E. Lash, A. Heguy, J. A. Eastham, H. I. Scher, V. E. Reuter, P. T. Scardino, C. Sander, C. L. Sawyers, and W. L. Gerald. (2010). Integrative genomic profiling of human prostate cancer. *Cancer Cell* 18 (1):11-22.

Tomlins, S. A., R. Mehra, D. R. Rhodes, X. Cao, L. Wang, S. M. Dhanasekaran, S. Kalyana-Sundaram, J. T. Wei, M. A. Rubin, K. J. Pienta, R. B. Shah, and A. M. Chinnaiyan. (2007). Integrative molecular concept modeling of prostate cancer progression. *Nat Genet* 39 (1):41-51.

Vanaja, D. K., J. C. Cheville, S. J. Iturria, and C. Y. Young. (2003). Transcriptional silencing of zinc finger protein 185 identified by expression profiling is associated with prostate cancer progression. *Cancer Res* 63 (14):3877-82

Varambally S, Yu J, Laxman B, Rhodes DR, Mehra R, Tomlins SA, Shah RB, Chandran U, Monzon FA, Becich MJ, *et al*. (2005). Integrative genomic and proteomic analysis of prostate cancer reveals signatures of metastatic progression. *Cancer Cell 8*, 393-406.

Wang, Y., Wang, Y., Liu, Q., Xu, G., Mao, F., Qin, T., Teng, H., Cai, W., Yu, P., Cai, T.*, et al.* (2014). Comparative RNA-seq analysis reveals potential mechanisms mediating the conversion to androgen independence in an LNCaP progression cell model. Cancer Lett *342*, 130-138.

Wallace, T. A., R. L. Prueitt, M. Yi, T. M. Howe, J. W. Gillespie, H. G. Yfantis, R. M. Stephens, N. E. Caporaso, C. A. Loffredo, and S. Ambs. (2008). Tumor immunobiological differences in prostate cancer between African-American and European-American men. *Cancer Res* 68 (3):927-36.

Welsh, J. B., L. M. Sapinoso, A. I. Su, S. G. Kern, J. Wang-Rodriguez, C. A. Moskaluk, H. F. Frierson, Jr., and G. M. Hampton. (2001). Analysis of gene expression identifies candidate markers and pharmacological targets in prostate cancer. *Cancer Res* 61 (16):5974-5978.

Yan L, Tian L, and Liu, S. (2015). Combining large number of weak biomarkers based on AUC. Stat Med. *34*, 3811-3830

Yu, Y. P., D. Landsittel, L. Jing, J. Nelson, B. Ren, L. Liu, C. McDonald, R. Thomas, R. Dhir, S. Finkelstein, G. Michalopoulos, M. Becich, and J. H. Luo. (2004). Gene expression alterations in prostate cancer predicting tumor aggression and preceding development of malignancy. *J Clin Oncol* 22 (14):2790-2799.

Zaehres, H., Lensch, M.W., Daheron, L., Stewart, S.A., Itskovitz-Eldor, J., and Daley, G.Q. (2005). High-efficiency RNA interference in human embryonic stem cells. Stem Cells *23*, 299-305.

Zhang D, Park D, Zhong Y, Lu Y, Rycaj K, Gong S, Chen X, Liu X, Chao HP, Whitney P, *et al*. (2016). Stem cell and neurogenic gene expression profiles link prostate basal cells to aggressive prostate cancer. Nat Commun 7:10798.

Zhang, Y., Liu, T., Meyer, C.A., Eeckhoute, J., Johnson, D.S., Bernstein, B.E., Nusbaum, C., Myers, R.M., Brown, M., Li, W.*, et al.* (2008). Model-based analysis of ChIP-Seq (MACS). Genome Biol *9*, R137.

SUPPLEMENTAL FIGURE LEGENDS

Figure S1. NANOG Protein Expression in Patient CRPC Samples

(A) IHC staining for NANOG using the R & D anti-NANOG antibody (Supplementary Table S1) in pLVX (vector control) or NP8-overexpressing LNCaP AI tumors grown in castrated NSG mice. Original magnification, x200.

(B) IHC staining for NANOG using the two indicated antibodies in a TMA containing 20 CRPC samples. Shown are 4 examples each of CRPC cores stained with Kamiya (against the N-ter) or with the R&D (against the C-ter) anti-NANOG antibodies. Shown at the bottom panels are 2 similarly stained benign prostate tissues (included in the same TMA) that showed negative NANOG staining. Original magnifications, x100; insets; x400.

Figure S2. NANOG Genomic Occupancy in LNCaP Cells

(**A-B**) UCSC genome browser traces of NANOG (or IgG) ChIP-Seq for promoters (boxed) occupied by both NANOG1 (N1) and NP8 (**A**) or preferentially by NP8 (**B**). pLVX refers to control cells expressing the empty vector used in NANOG ChIP-Seq analysis.

(**C-D**) Cistrome binding of NP8 versus androgen receptor (AR), FOXA1 and NKX3.1 (**C**) or versus pLVX (**D**). AR, FOXA1, and NKX3.1 data are from Tan et al, 2012 (GEO: GSE28264).

Figure S3. NANOG Occupancy of the PCa Genome Correlates with Steroid Hormone Transcription Factor Occupied Loci and Is Enriched in Active Chromatin

(A) Motif analysis by MEME of NANOG1 occupied chromatin in LNCaP cells reveals FOXA1 motif as the most abundantly occupied site (i.e., 689 sites/800 peaks; the E-value for the occurrence of this motif =9.5e-266).

(B) Signal distribution heat map analysis of ChIP-Seq peaks (NP8, AR, FOXA1, and NKX3.1) centered on NP8, +/- 10 kb from the peak (total 14,449 peaks), sorted according to NANOG peak tag intensity and grouped according to the following classifications: 1) 4-factor common (3,790; 26%), 2) minus NKX3.1 (4,684; 32%), 3) minus FOXA1 (244; 1.6%), 4) minus AR (34; <1%), 5) NP8 and AR only (1,587; 11%), 6) NP8 and FOXA1 only (982; 6.8%), 7) NP8 and NKX3.1 only (70, <1%) and 8) NP8 only (3,058; 21%).

(**C**) Bar chart showing the proportion of NP8 binding sites co-occupied by FOXA1, AR and/or NKX3.1 in the absence of androgen.

(D) Bar chart showing the proportion of the steroid-hormone receptor complex component (AR, FOXA1, and/or NKX3.1) co-occupied by NP8 in the presence (AD) or absence (AI) of androgen.

(E) Distribution of histone marks +/- 10 Kb around NP8 ChIP-Seq peaks occurring among promoter region (within -8 Kb to +2 Kb relative to TSS) associated, NANOG-occupied chromatin regions. H3K4me1, H3K4me3 and H3K27me3 ChIP-Seq was performed in our lab (Liu et al., manuscript in preparation) while the H3K4me2 data was from the published literature (GSE20042:GSM503905). RPKM, reads per kilobase of transcript per million mapped reads.

Figure S4. NANOG Interacts with AR and FOXA1

(A) CentriMo analysis of the positional distribution of motif 2 (shown on top) corresponding to the consensus sequence of NFI-family. Random 600 peaks (MACSe-10) +/- 500 bp of the pinnacle were analyzed.

(B) Analysis of protein-protein interactions via Duolink Proximity Ligation Assay (PLA; Sigma) using anti-NANOG (Cell Signaling, rabbit mAb D73G4) or anti-FOXA1 (positive control; Abcam, goat pAb) with anti-AR (Santa Cruz, mouse mAb) antibodies. Confocal optical sections (100X objective; 1 μm z-stacks) were integrated into a composite image. Green dots indicate protein-protein interactions within ~40 nm ranges. Similar PLA with NANOG and FOXA1 did not result in meaningful data (data not shown).

(C-E) NP8 binds the FOXA1 consensus DNA motif. EMSA was performed as described in SEP using biotinylated FOXA1 motif in the *UBE2C* gene promoter as the probe (lanes 1). C and D represent two independent experiments whereas E represents a separate experiment in which an increasing ratio of FOXA1 over NP8 (lanes 6-9) was used. Note that the cold unlabeled probes significantly reduced binding of FOXA1 (lanes 3) or NP8 (lanes 5) binding to the biotinylated probe.

Figure S5. Gene Expression Changes Induced by NP8 (or NANOG1) in RNA-Seq Analysis.
Shown is the heat map presentation of differentially expressed genes (DEGs). Hierarchical clustering was performed on DEGs from any of the five comparisons (NANOG1/NP8 vs. pLVX under AD/AI and short/long term) using the log2 ratio values, which, in each row, were rescaled so that the sum of the squares of the values is 1.0. Euclidean distance and ward clustering method were used to construct the dendrogram, which classified the genes into 11 groups (marked on the left by different color-coded bars). Shown on the right are 6 clusters of genes that displayed distinct patterns of changes.

Figure S6. NANOG Induces Distinct Gene Expression Changes that Correlate with Castration Resistance and Patient Survival.

(A-B) GSEA of Cluster 1 (A) or Cluster 1&3 (B) genes showing their enrichment in normal human prostate differentiated luminal cells (a), differentiated (PSA^+) LAPC9 cells (b), (differentiated) adenocarcinomas (T) in comparison to metastases (c), AD LNCaP cells in comparison to AI LNCaP cells (d), AD LAPC9 tumors in comparison to AI LAPC9 tumors (e), AD LNCaP tumors in comparison to AI LNCaP tumors (f), and patient tumors before ADT vs. after ADT (f).

(C) IPA Upstream Regulator analysis of the 258 genes in Clusters 1&3 reveal numerous genes as downstream targets of the actions of androgens, dihydro-testosterone, and R1881 (metribolone).

(D) Integrative analysis of NP8 genomic occupancy (ChIP-Seq) and NANOG induced DEG clusters. The analysis was performed by Fisher's Exact test to determine the enrichment of DEGs co-occupied by NP8 with AR and/or FOXA1 within a +/- 10 Kb window of each peak.

(**E-F**) *Oncomine* Concept Analysis showing the under-expression of 20 Cluster 1&3 genes in prostate tumors (T) compared to normal (N) tissues (E) and of 20 genes in PCa metastasis (M) in comparison to primary tumors (F).

(**G-H**) Survival analysis links Cluster 1&3 genes to better patient survival. A 33-gene signature from Cluster 1&3 genes (see SEP) was used to stratify PCa patient survival in the Setlur dataset (see SEP for *Oncomine* data sets), in which patients with higher expression of the signature had significantly better overall survival (i.e., low risk of dying) than those with lower expression of the signature (G). The same signature also predicts for better patient survival in a testing data set (H). In Cluster 1&3, higher expression corresponds to lower risk.

(**I-J**). GSEA of Cluster 2 (I) and Cluster 6 (J) genes showing their enrichment in undifferentiated (PSA^-) LNCaP cells (a), LNCaP AI cells (b), and patient tumors after ADT vs. before ADT (c).

(**K**) GSEA shows that DEGs induced in NP8 AI d22 cells are enriched in GO terms "Cell Cycle" and "DNA Replication".

(**L**) *Oncomine* Concept Analysis showing the overexpression of 20 Cluster 5 genes in prostate tumors

(T) compared to normal (N) tissues (left) and of 20 Cluster 5 genes in PCa metastasis (M) in comparison to primary tumors (right).

Figure S7. NP8 Dynamically Upregulates Cell Motility Genes and Engages the MYC Transcriptional Program

(A-D) IPA biological function profiling of DEGs (up >1.5X, *P*<0.05) in NP8-overexpressing cells relative to pLVX controls in androgen-dependent (AD) d5 (**A**) and d12 (**B**) and androgen-independent (AI) d7 (**C**) and d22 (**D**) conditions. Several major and relevant biological pathways were highlighted in red.

(E-F) 'Cell movement' genes are highly enriched in LNCaP cells expressing either NANOG1 (N1) or NP8 under AD or AI conditions for various periods of time. Shown in E is a bar graph depicting the total number of "Cellular Movement" related genes upregulated by NANOG (>1.5X, *P*<0.05) in the "Cell Movement" category of the IPA Biological Processes, with the associated p-values indicated. Shown in F is the Venn presenting overlap of movement-related DEGs (from E) in each group as indicated.

(**G**) Heat map presentation of "Cell Movement" genes (~120) upregulated (>1.5X and *P*<0.05 in at least one group) in IPA Biological Processes analysis of IPA.

(H) Western blot showing upregulation of c-MYC protein in NANOG-expressing LNCaP cells treated with MDV3100 (10 μM) for 5 or 12 days.

(**I**) IPA Upstream Regulator analysis implicates MYC and FOXM1 as potential downstream mediators of NANOG signaling as evidenced by multiple MYC and FOXM1 transcriptional targets (many of which overlapped) upregulated in NANOG-expressing cells.

Figure S8. NANOG Occupancy of Genomic Loci and Upregulation of UBE2C Protein Under AI Conditions.

(A) Schematic of primer design for ChIP-qPCR (Fig. 7B-C) targeting NANOG occupied loci relative to the position of the putative target gene (TSS indicated by an arrow). Forward (F) and reverse (R) primer sequences are shown in Table S7 targeting the indicated ChIP-Seq peak occurring in a distal enhancer (Enh) or promoter (Prom, -8 Kb to +1 Kb) region.

(B) Proliferation assay of LNCaP cells overexpressing NANOGP8 (*vs* pLVX) cultured in 20 μM MDV3100 for 30 d. EdU pulsed cells were detected via immunofluorescence staining and quantified by flow cytometry. Shown are representative histograms from one experiment (n=3).

(C) Western blot analysis showing regulation of UBE2C protein in LNCaP cells expressing NANOG1 (N1) or NANOGP8 (NP8) vs pLVX control cells. Cells were cultured in androgen-independent (AI) conditions using charcoal-dextran stripped serum (CDSS) for the indicated time.

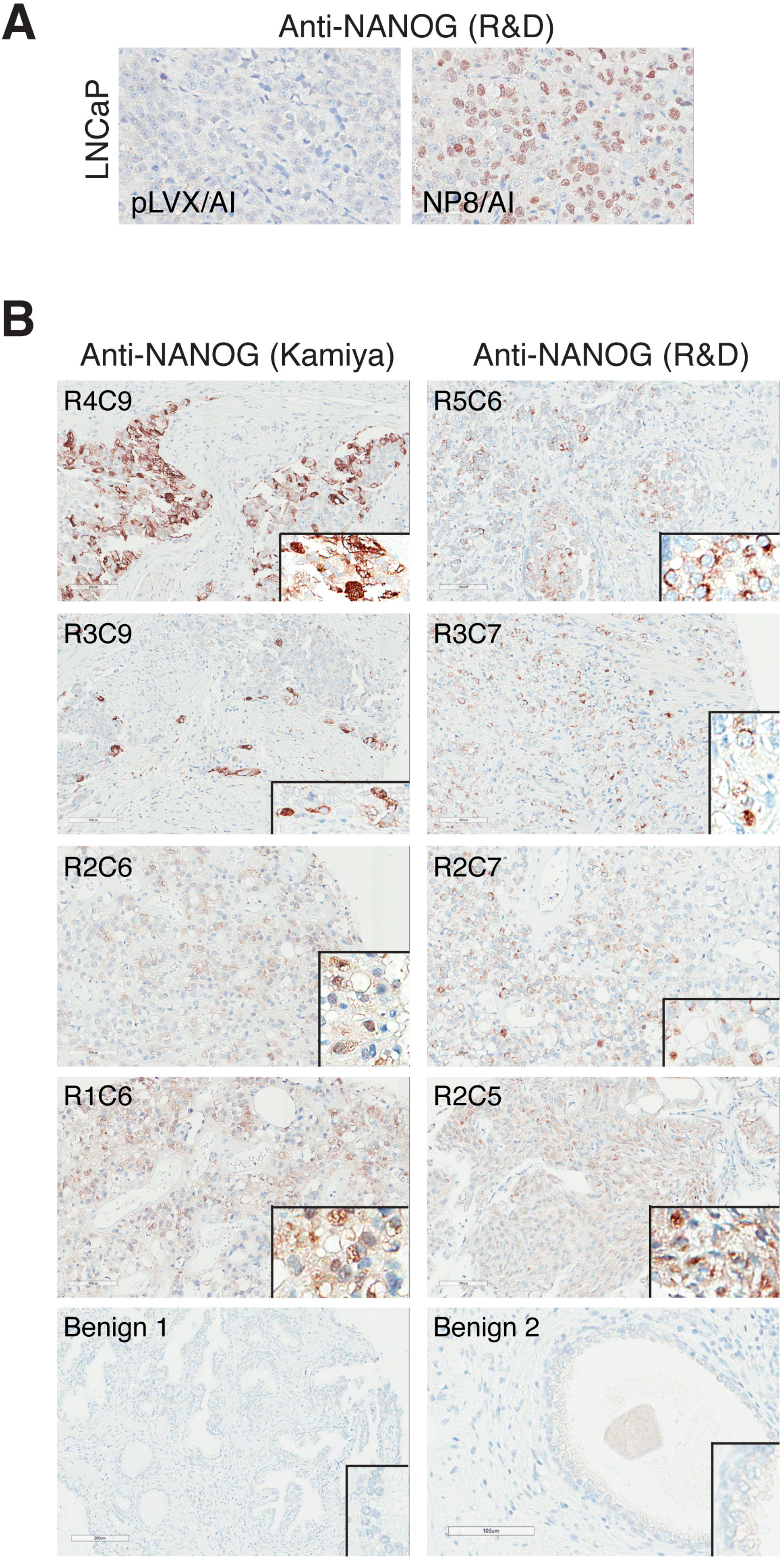

Jeter et al. Supplemental Figure 1

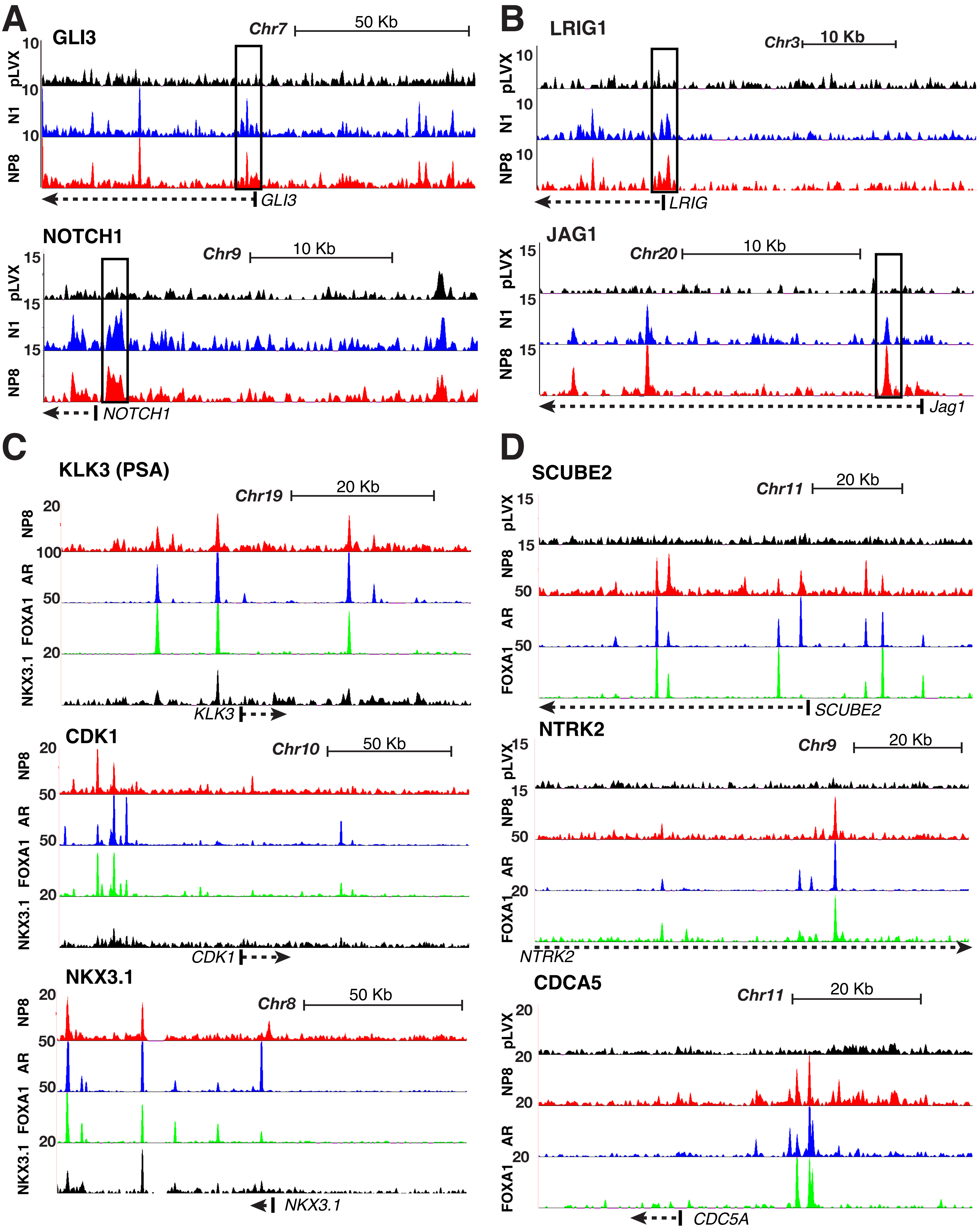
A
GLI3
Chr7
50 Kb
pLVX
N1
NP8
GLI3
NOTCH1
Chr9
10 Kb
NOTCH1
B
LRIG1
Chr3
10 Kb
LRIG
JAG1
Chr20
Jag1
C
KLK3 (PSA)
Chr19
20 Kb
AR
FOXA1
NKX3.1
KLK3
CDK1
Chr10
CDK1
NKX3.1
Chr8
D
SCUBE2
Chr11
SCUBE2
NTRK2
Chr9
NTRK2
CDCA5
CDC5A

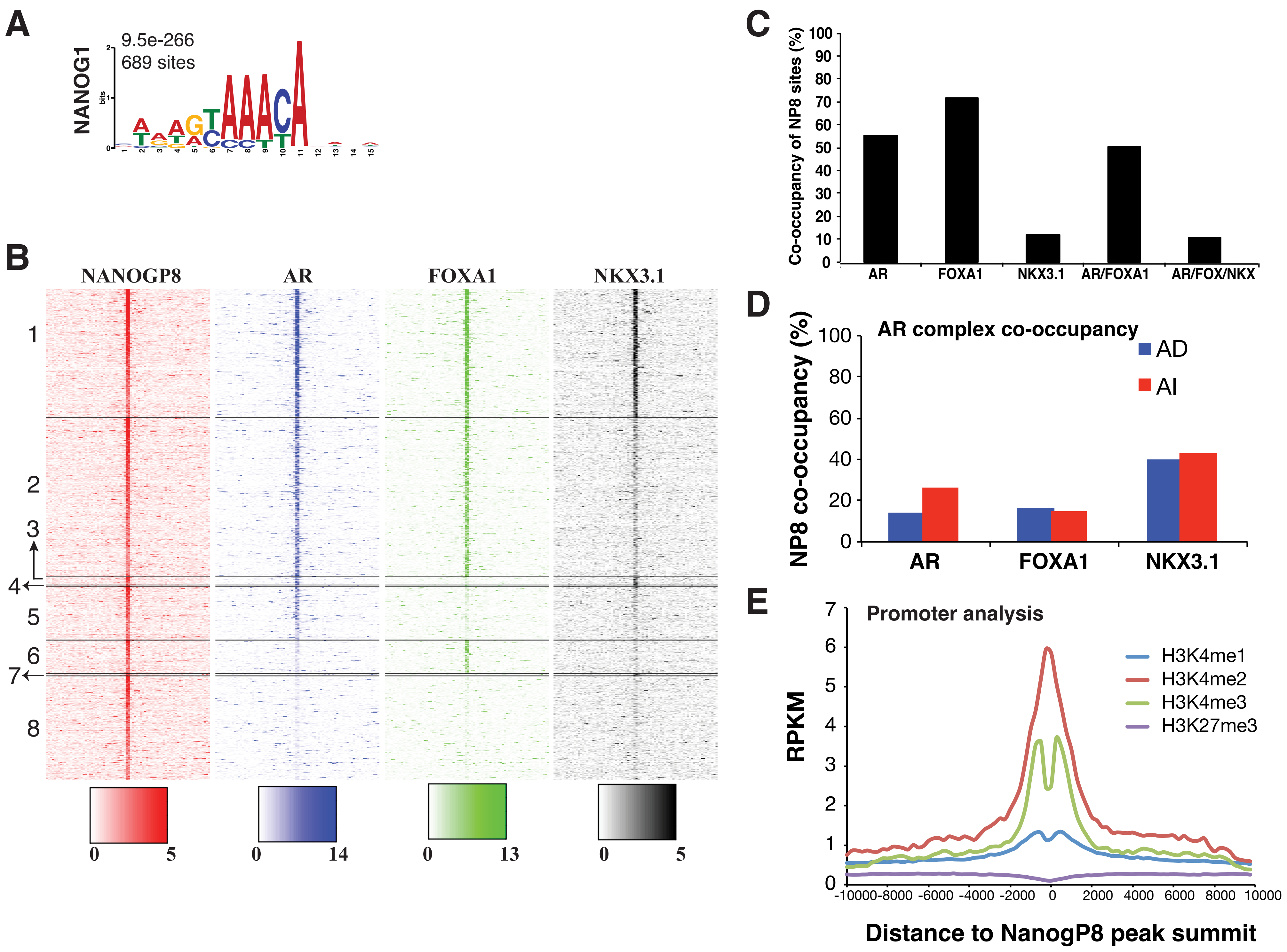

Jeter et al., Supplemental Figure 3

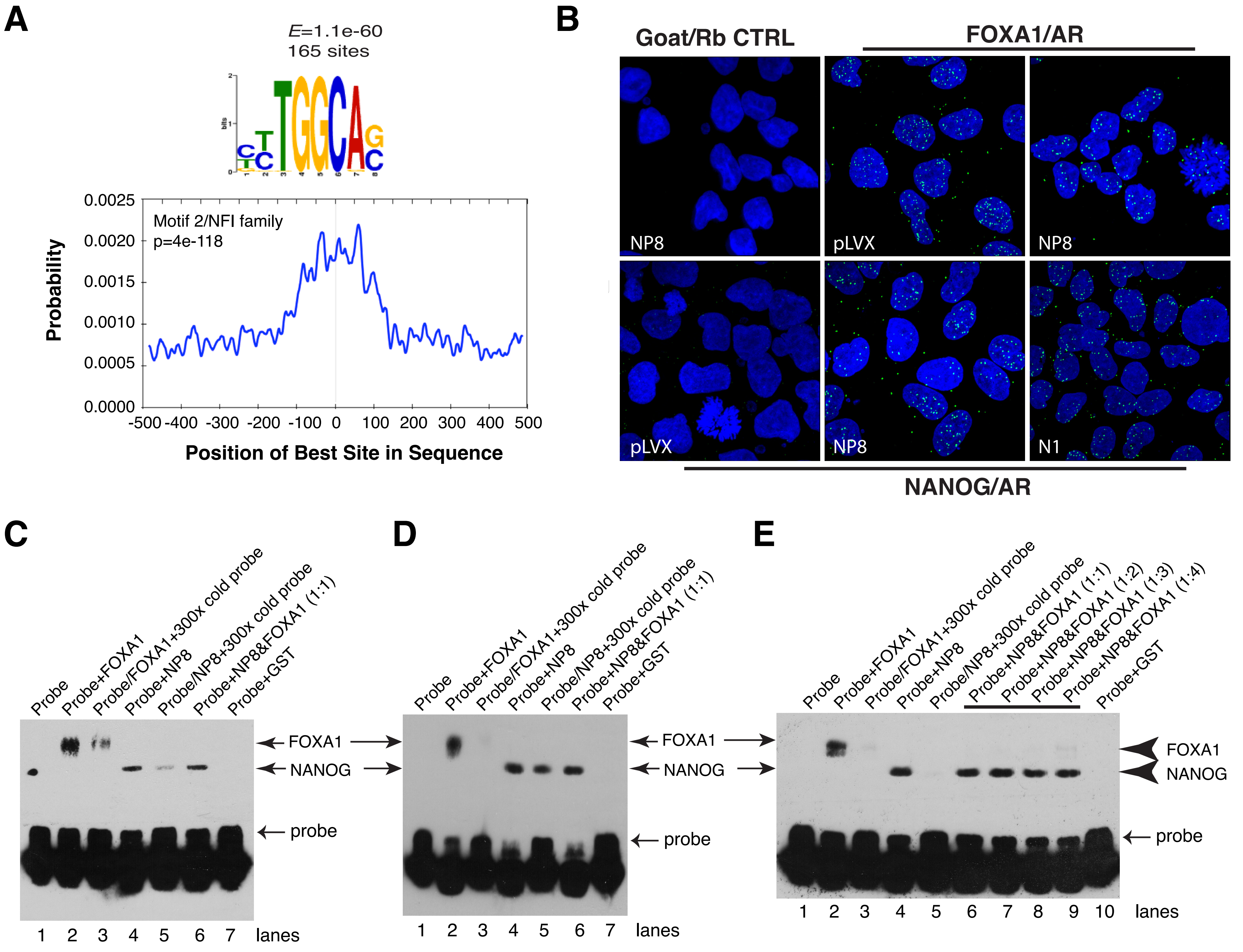

Jeter et al. Supplemental Figure 4

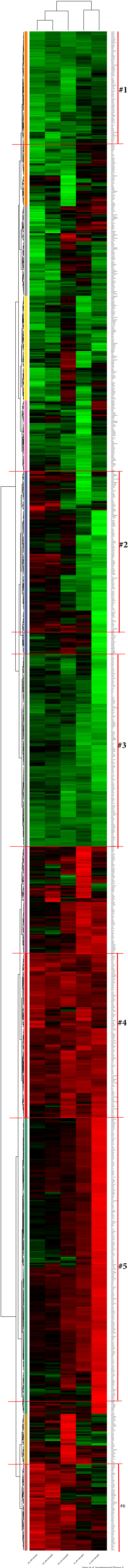

Jeter et al, Supplemental Figure 5

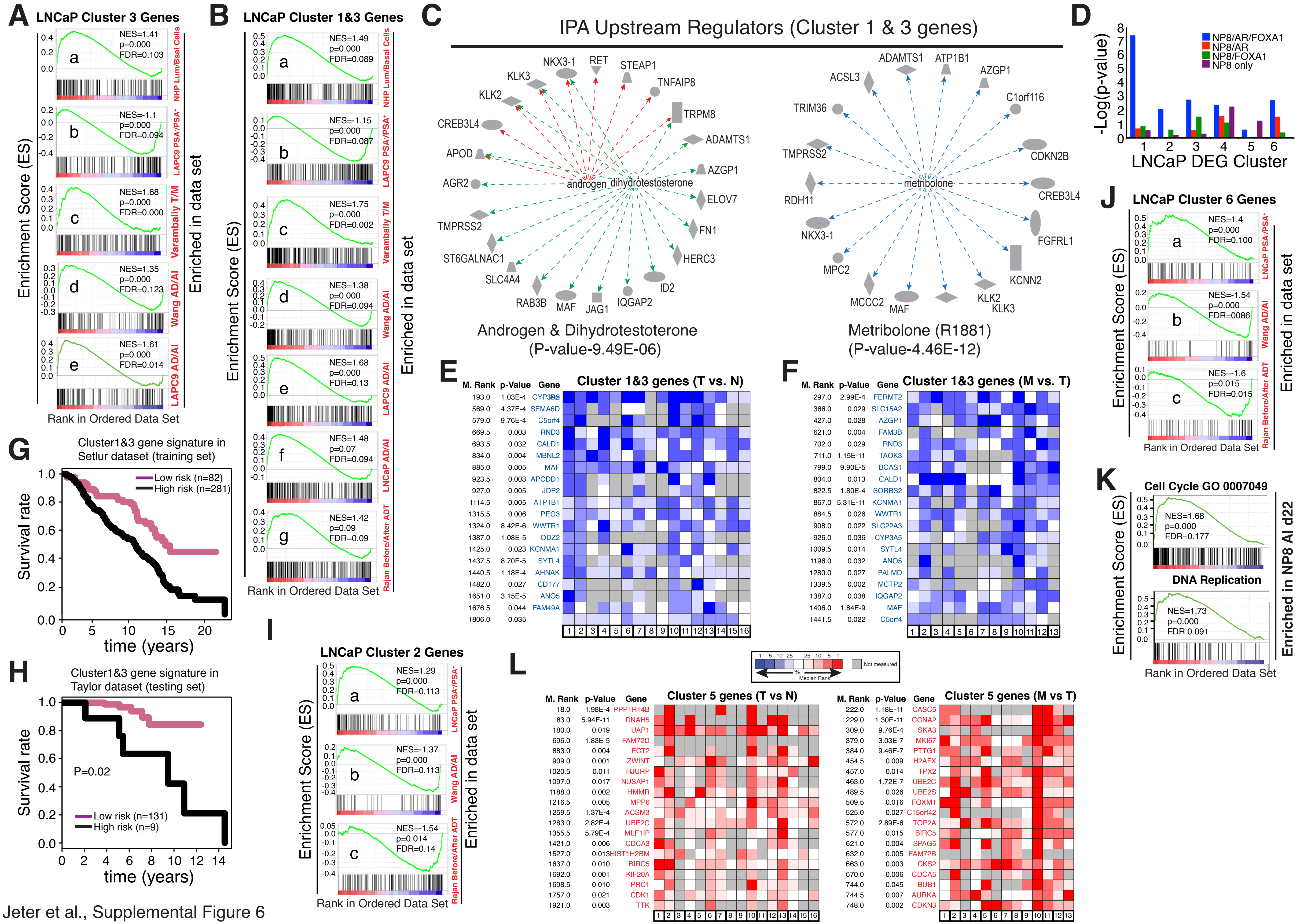

Jeter et al., Supplemental Figure 6

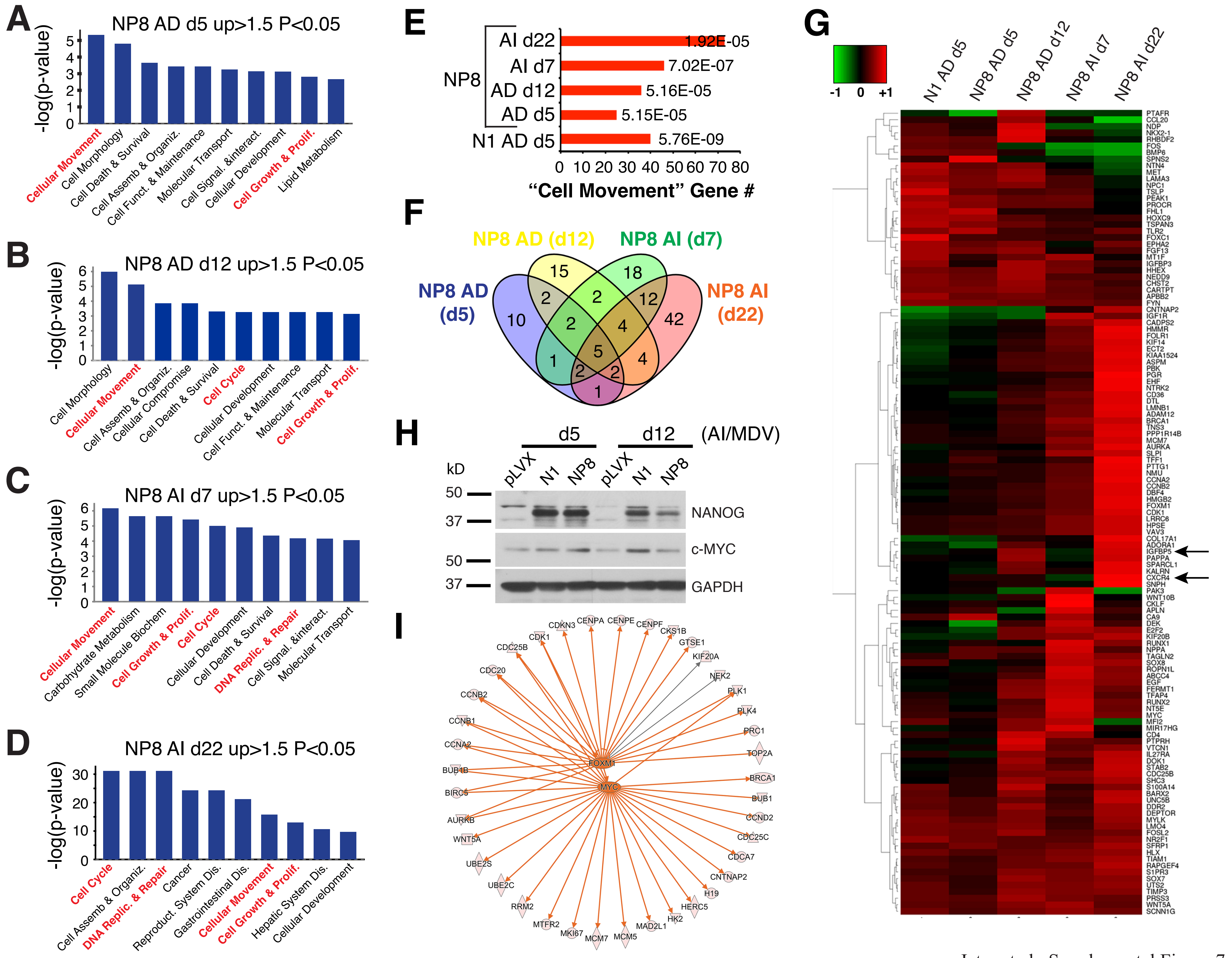

Jeter et al., Supplemental Figure 7

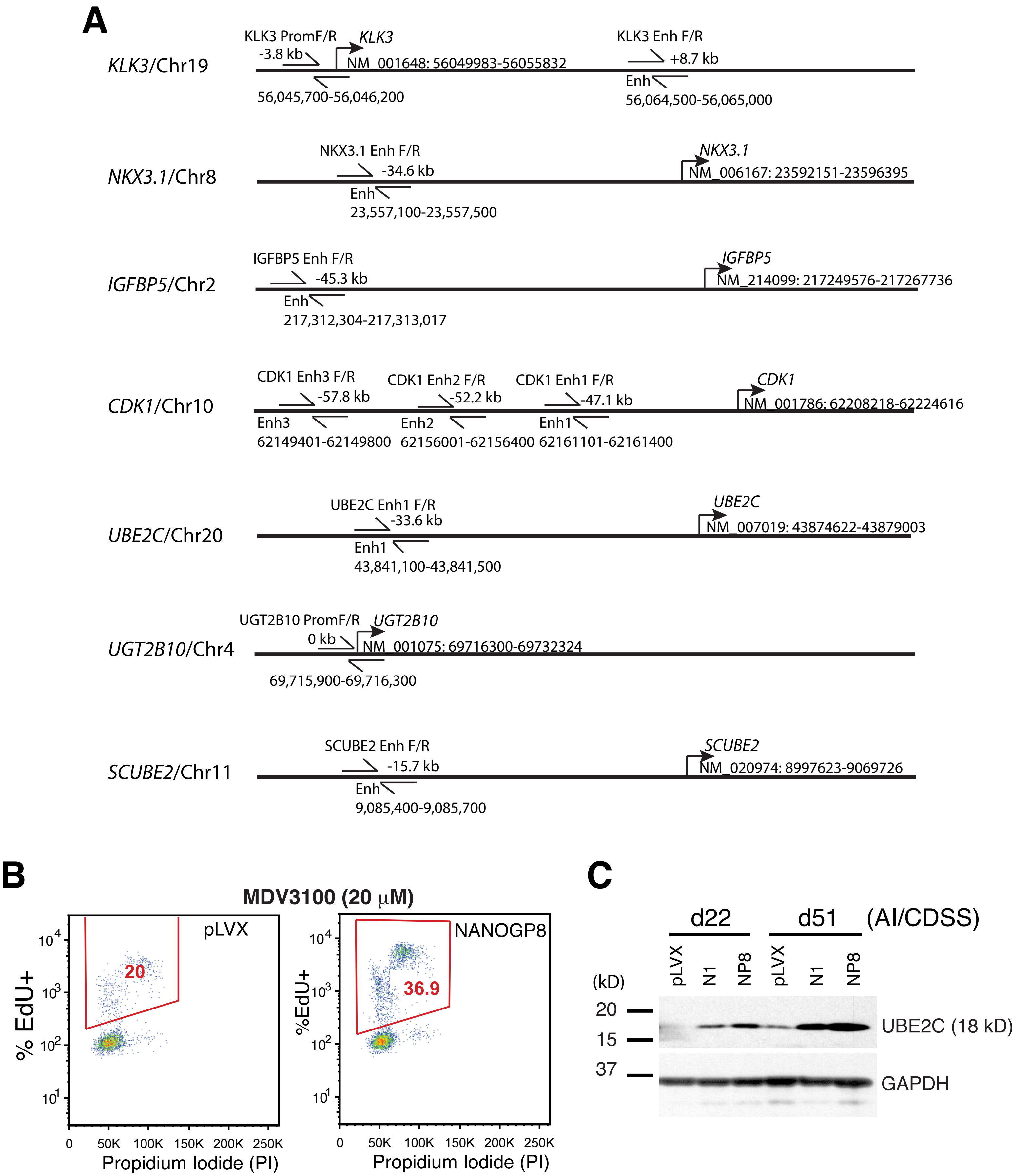

Jeter et al., Supplemental Figure 8

Table S1. Primary antibodies used in this study.

Protein	Company	Cat# (clone)	Host/type	Application
FOXA1	Abcam	ab5089	Goat polyclonal	IP, IF, WB
FOXA1	Abcam	ab23738	Rabbit polyclonal	IP, IF, WB
AR	Santa Cruz	sc-816<br>sc-816G	Rabbit polyclonal<br>Goat polyclonal	WB
AR	Santa Cruz	sc-7305<br>(441)	Mouse monoclonal	IF, WB
NANOG	R&D	AF1997	Goat polyclonal	IHC, IP, ChIP, WB, PLA
NANOG	Kamiya	PC-102	Rabbit polyclonal	IHC, WB
NANOG	Cell Signaling	4903S<br>(D73G4)	Rabbit monoclonal	WB, IHC, IF
NANOG	Cell Signaling	5232<br>(D73G4)	Rabbit monoclonal	IF, IP, PLA
NANOG	Santa Cruz	sc-33759	Rabbit polyclonal	ChIP-Seq
Normal IgG	Santa Cruz	sc-2027	Rabbit	IP
Normal IgG	Santa Cruz	sc-2028	Goat	IP
KLK3 (PSA)	Dako	M0750	Mouse monoclonal	WB
MYC	Santa Cruz	sc-40	Mouse monoclonal	WB
SCUBE2	Aviva Systems	OAAB07706	Rabbit polyclonal	WB
S1PR3	Santa Cruz	SC-22211	Goat polyclonal	WB
UBE2C	Boston Biochem	A650	Rabbit polyclonal	WB
NKX3.1	Santa Cruz	sc-15022	Goat polyclonal	WB
NKX3.1	Abcam	ab78008	Rabbit polyclonal	WB
GAPDH	Santa Cruz	sc-25778	Rabbit polyclonal	WB

Abbreviations: ChIP, chromatin-immunoprecipitation; IF, immunofluorescence; IgG, immunoglobulin; IP, immunoprecipitation; PLA, proximity-ligation assay; WB, Western blot.

Table S7: ChIP-qPCR SYBR primers used in this study.

Presumed Target	Chr #	NANOG peak	Approx. Distance (Kb)	F primer	R primer
***UBE2C* Enh1**	20	43,841,100-43,841,500	-34	TGCCTCTGAGTAGGAACAGGTAAGT	TGCTTTTTCCATCATGGCAG
***CDK1* Enh1**	10	62,161,100-62,161,400	- 47	ACAGTCCAAATTCAAAGCTCACTT	GCTACTAATATCTGGAGGGCCAA
***CDK1* Enh2**	10	62,156,000-62,156,400	-52	AAGTGGACACAAGGTTCAACAG	AATGCACATAGAGCACTTGGTA
***CDK1* Enh3**	10	62,149,400-62,149,800	-58	TGGCATTGATTTTGGTGCAGT	GCAGCTAAAGTCTATGAAGATTCCA
***KLK3* Prom**	19	56,045,700-56,046,200	-4	TGGGACAACTTGCAAACCTG	CCAGAGTAGGTCTGTTTTCAATCCA
***KLK3* Enh**	19	56,064,500-56,065,000	+8.7	CTGTTCACCCAGAGCCTTCC	TGAAGCCAGATGTGGTAGACAC
***NKX3.1* Enh**	8	23,557,101-23,557,500	-35	AGTTCAACCCTCTGGCTTGG	AAGTGCCTCCCTCCTAGCTG
***SCUBE2* Enh**	11	9,085,400-9,085,700	- 16	ACTGAGTACACTGACAAAAATCCAA	CACCACCAATGTTTGAAATGCC
***UGT2B10* Prom**	4	69,715,900-69,716,300	0	GCAGGACTCTCTCACTTGC	ATCCACCTAAGTTTAATGACCTTGC
***IGFBP5* Enh**	2	217,312,304-217,313,017	-45	TGGGTGCAACAATGTAGGTACG	AAGGGGGAATAAGGACGGGT
***CDCA5* Enh1**	11	64,626,193-64,626,726	-18	CAGCGACAATGACAGCACAC	GCTACCTCAAGAAGGAAGCTTGA
***CDCA5* Enh2**	11	64,628,209-64,629,010	-20	TTCTCGTTTCTCTGCTGGGG	CCAGATGTCCAGGGGTGTTG

*Primers for qPCR (SYBR) designed against sequence at indicated position (hg18) on chromosome as shown, demarcating central region of NANOG peak upstream (minus) or downstream (plus) from the transcription start site (TSS) or transcription end site (TES), respectively. Abbreviations: Chr, chromosome; Enh, enhancer; F, forward; Prom, promoter; R, reverse.
